# Droplet impact on doubly re-entrant structures

**DOI:** 10.1038/s41598-024-52951-2

**Published:** 2024-02-01

**Authors:** Navdeep Sangeet Singh, Thanaphun Jitniyom, Miguel Navarro-Cía, Nan Gao

**Affiliations:** 1 School of Engineering, University of Birmingham, Birmingham, B15 2TT UK; 2 School of Physics and Astronomy, University of Birmingham, Birmingham, B15 2TT UK

**Keywords:** Mechanical engineering, Fluid dynamics, Surfaces, interfaces and thin films

## Abstract

Doubly re-entrant pillars have been demonstrated to possess superior static and dynamic liquid repellency against highly wettable liquids compared to straight or re-entrant pillars. Nevertheless, there has been little insight into how the key structural parameters of doubly re-entrant pillars influence the hydrodynamics of impacting droplets. In this work, we carried out numerical simulations and experimental studies to portray the fundamental physical phenomena that can explain the alteration of the surface wettability from adjusting the design parameters of the doubly re-entrant pillars. On the one hand, three-dimensional multiphase flow simulations of droplet impact were conducted to probe the predominance of the overhang structure in dynamic liquid repellency. On the other hand, the numerical results of droplet impact behaviours are agreed by the experimental results for different pitch sizes and contact angles. Furthermore, the dimensions of the doubly re-entrant pillars, including the height, diameter, overhang length and overhang thickness, were altered to establish their effect on droplet repellency. These findings present the opportunity for manipulations of droplet behaviours by means of improving the critical dimensional parameters of doubly re-entrant structures.

## Introduction

Liquid repellent surfaces can be used to improve many industrial applications or processes. These include self-cleaning coatings^1,2^, drag reduction^3,4^, anti-icing surfaces^5,6^, enhancement of boiling^7,8^ and condensation^9,10^. In practice, liquid repellent surfaces can be realised by chemically modifying micro-structured substrates, such as those consisting of pillars, with low-surface-energy coatings. Conventional pillars are typically straight, that is, having a vertical sidewall perpendicular to the interface. However, the liquid repellence of regular pillars is largely limited to superhydrophobicity. Tuteja et al. fabricated re-entrant structures by introducing a horizontal overhang structure on top of the straight pillars parallel to the interface^11^. This allowed the surface to become omniphobic, where the surface can repel both high- and low-surface-tension liquids. Liu et al. then further made doubly re-entrant structures by adding an additional vertical overhang to the re-entrant structure^12^. As a result, the structures were able to resist fluorinated solvents, including perfluorohexane (C_6_F_14_) that has a low surface tension value of 10 mN/m.

The concept behind the superior liquid repellency of the doubly re-entrant structure is related to manipulating the interfacial surface tension forces to be perpendicular against the direction of droplet penetration or impact. In other words, the vertical overhang provides a maximal suspension force compared to re-entrant or straight pillars^12^, suspending the liquid from wicking further into the cavities. Nonetheless, a sufficiently small solid fraction (~ 6%) is required to maintain superomniphobicty against the wetting liquids and large external pressures^12^. However, there have been many variants of the doubly re-entrant design. For example, Yun et al. created hierarchical surfaces where doubly re-entrant pillars were applied on a micro-wrinkled surface^13^, inspired by the inherent surface topography of springtail cuticles^14^. It was noted that the presence of the doubly re-entrant pillars on top made the surface resistant to elevated external pressure, enabling the repellency of much faster liquid droplets with low surface tension, with the Weber number (*We*) up to 287. The Weber number is known as the ratio of the inertial force to the surface tension force:

1 $$\begin{array}{c}We=\frac{{\rho }_{l}{V}^{2}{\lambda }_{c}}{{\gamma }_{lg}},\end{array}$$

where ${\rho }_{l}$ is the density of the liquid (kg/m^3^), *V* is the velocity of the liquid (m/s), ${\gamma }_{lg}$ is the liquid–gas interfacial surface tension (N/m) and ${\lambda }_{c}$ is the hydrodynamic length of the fluid of interest, in this case, equal to the diameter of the droplet (m). Sun et al. 3D printed hierarchical doubly re-entrant pillars consisting of multiple layers of doubly re-entrant overhangs^15^. The notion behind the design was to create additional pressure barriers, in order to prevent the droplet from further penetrating the cavity. This was accomplished by increasing the diameter of the overhangs along the length of the pillar, which consequently reduced the pitch of the cavity at each layer. The overall breakthrough pressure was reported to be much higher than single layered doubly re-entrant structures as the hierarchical surface was able to repel water droplets at Weber numbers that were larger than 25. Here, the breakthrough pressure is defined as the maximum suspension pressure generated by the liquid surface tension that is acting around the perimeter of the pillars across the liquid–gas interface^12,16,17^. For the pillars to suspend the liquid, this maximum pressure must be greater than or equal to the downward directed hydrostatic (for static droplets) or dynamic (for impacting droplets) pressures.

In addition, Liu et al. utilised triply re-entrant structures by allocating an additional horizontal overhang to the vertical one of the doubly re-entrant structure^18^. However, the additional overhang structures did not improve the droplet repellency (contact angle hysteresis) over the previously reported doubly re-entrant structures. On the other hand, surfaces consisting of isolated doubly re-entrant cavities have been reported to obtain more robust liquid repellency than those consisting of doubly re-entrant pillars in various scenarios, including droplet impact^19,20^. However, as the solid fractions of these cavities are higher than those of doubly re-entrant pillars, the apparent contact angle is reduced, causing the surface to partially lose their superhydrophobicity. Particularly, the contact angle hysteresis of doubly re-entrant cavities can be greater than 30°, significantly higher than that of doubly re-entrant pillars^19,20^. This is because some of the liquid will be trapped within the cavities as the liquid recedes, especially during capillary condensation.

It should also be noted that, while doubly re-entrant structures have been demonstrated to be superior, there is still a lack of quantitative investigations into the effect of the design parameters on the liquid repellency of the surface. Numerical simulations can provide such insight by depicting the principal hydrodynamic aspects that are involved during droplet impact, although there are limited successful studies related to numerical simulations of droplet impact on doubly re-entrant structures. In fact, most numerical studies examine droplet impact on plain or macro-scale surfaces^21–24^. Recently, a study by Hu et al. simulated droplet impact on straight superhydrophobic micro-structured pillars^25^. Their investigation included a parametric analysis of droplet impact behaviours as a function of droplet velocities, intrinsic contact angles and pillar heights, noting whether the droplet rebounded or flooded the surface. A numerical study by Panter et al. conducted a parametric analysis of re-entrant and doubly re-entrant structures against various breakthrough pressures^26^. However, the relevant numerical model did not include droplet repellency.

Here, we further extend the numerical study of droplet impact towards doubly re-entrant pillars and compare the simulations against experimental results to investigate the fundamental phenomena. We show that the behaviours of the impacting droplet can be manipulated by altering the dimension and pitch of the doubly re-entrant pillars. Further, hydrophobizing the pillars enables the droplet to bounce off the surface at a faster rate. Nonetheless, once the impacting pressure exceeds the breakthrough pressure, the droplet will flood the interstices of the doubly re-entrant pillars regardless of being hydrophobized. This leads to further questions of how the design parameters of the doubly re-entrant pillars, including the length and thickness of the vertical overhang, can be optimised in order to prevent the meniscus from touching the bottom of the surface. Through analysing the interfacial pressure and velocity contours plots, we illustrate the regions of significance that play an important role in droplet repellency.

## Methodology

**Figure 1:**
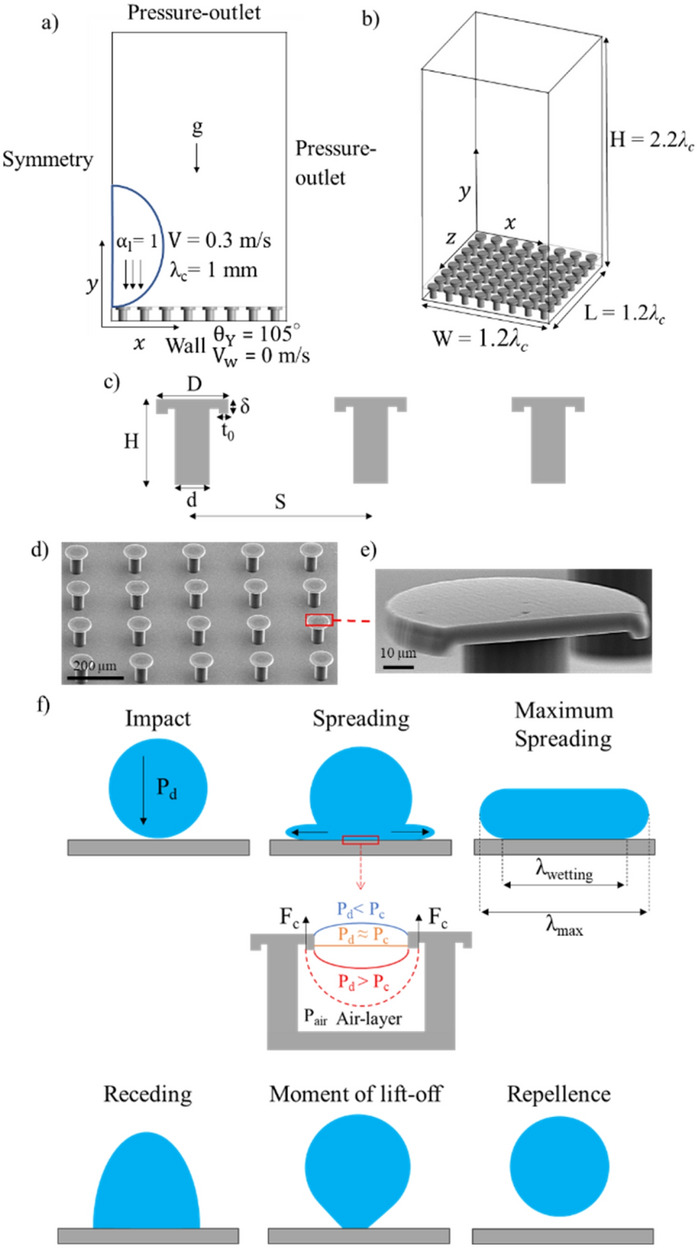
Fluid domain design with prescribed boundary and initial conditions: (**a**) side view and (**b**) iso view. (**c**) Key dimensions for doubly re-entrant pillars. (**d**) SEM image of the fabricated doubly re-entrant pillars, with D = 100 µm, H = 100 µm, S = 200 µm, t_o_ = 10 µm and δ = 10 µm. (**e**) Cross-sectional image of the doubly re-entrant pillar. (**f**) Schematic illustration of the droplet impact process, where P_d_ is the total droplet impact pressure (Pa), F_c_ and P_c_ are the capillary force (N) and pressure (Pa) (see Supplementary Information, Sect. 5 for more details), P_air_ is the air pressure within the air-layer (Pa), λ_max_ and λ_wetting_ are the maximum spreading diameter and wetting diameter of the droplet (m).

The arrangement of the fluid domain and boundary conditions is configurated (using ANSYS Fluent) as shown in Fig. 1a and b. A water droplet is initially placed at the XYZ axis containing a liquid volume fraction (*α*_*l*_) of one with a diameter of 0.8–1.2 mm (Fig. 1a). This is followed by an initial velocity condition applied on the droplet parallel to the direction of gravity which corresponds to a *We* = 1.25–1.7. As only a quarter of the simulation domain is used, a symmetry boundary condition is applied at YZ and XY planes where the droplet is placed. This particular setup is used to save computational resources, such as the number of mesh elements and computation time, as the flow is considered to be symmetric. Furthermore, a pressure-outlet condition is applied at the faces opposite to the position of the droplet to allow the liquid to escape either when it is spreading or bouncing. To simulate the ambient environment, the operating pressure chosen is equal to the atmospheric pressure (given as 101,325 Pa). A wall boundary condition is applied at the pillars, having an intrinsic contact angle of 70°, 105° and 120°, with a no-slip condition (*V*_*w*_ = 0 m/s). For millimetric sized drops, the slip velocity at the liquid–solid interface is deemed to be insignificant. The dimensions of the fluid domain (i.e. width, length and height) are dependent on the diameter of the droplet (Fig. 1b). This is to allow proper visualization of droplet impact behaviour as the liquid spreads, and rebounds towards the positive y-direction.

The properties of water employed in the simulation are isothermal (at ambient temperature), where *μ*_l_ = 1.003 mPa·s, *ρ*_l_ = 998.2 kg/m^3^, *ρ*_g_ = 1.225 kg/m^3^, *μ*_g_ = 0.018 mPa·s and *γ*_lg_ = 72 mN/m. *μ*_*l*_ is the dynamic viscosity of the liquid (Pa·s), *μ*_*g*_ is the dynamic viscosity of air (Pa·s) and *ρ*_g_ is the density of air (kg/m^3^). Additionally, the dimensional parameters to be configured for the pillars are displayed in Fig. 1c. The intrinsic contact angles used for each case are listed in the Supplementary Information (Sect. 1, Table S1). In order to compare the numerical simulations against the experimental results, the intrinsic contact angles are set to match the values (70° before being hydrophobized and 120° after being hydrophobized) of the photoresin which was used to fabricate the pillars via direct laser lithography. More information on the contact angle measurements can be seen in the Supplementary Information (Sect. 2, Table S2). Figure 1d and e display the SEM (scanning electron microscopy) images of the fabricated doubly re-entrant pillars and their cross section revealing the overhang structure. Details of fabrication can be seen within the Supplementary Information (Sect. 4). It is important to note that if the breakthrough pressure of the surface is greater than the total impacting pressure of the droplet, the droplet will be cushioned on a thin layer of air and emanate capillary waves instead of contacting the bottom of the cavity^27–29^. For functional surfaces having a composite interface, this air layer is present within the composite structure (Fig. 1f)^5^. With low Weber numbers, the air layer on a planar surface is considered to be incompressible. This is based upon the gas compressibility factor, *ε*, which must be much greater than one to ensure incompressibility^28,30^:

2 $$\begin{array}{c}\varepsilon =\frac{{P}_{a}}{\sqrt[3]{R{{\mu }_{g}}^{-1}{{\rho }_{l}}^{4}{V}^{7}}}\end{array}$$

where *P*_*a*_ is the ambient pressure of the air (Pa), and *R* is the radius of the droplet (m). In our case, the gas compressibility factor was calculated to be more than 40, which suggests the flow remain incompressible in the absence of any micro-pillars. Nonetheless, the air within the vicinity of the pillars’ cavities may become more compressible as air is trapped underneath the droplet.

Additionally, to prevent the hydrostatic force (i.e. the weight of the droplet) altering the hydrodynamics during impaction, the droplet diameter must be lower than the capillary length of the selected liquid. The capillary length is obtained when the hydrostatic pressure of the liquid is in equilibrium with the Laplace pressure:

3 $$\begin{array}{c}{\kappa }^{-1}=\sqrt{\frac{{\gamma }_{lg}}{{\rho }_{l}g}}\end{array}$$

where *g* is the gravitational acceleration.

From the given fluid properties at ambient temperature, the capillary length for water is calculated as 2.7 mm. Therefore, based on the calculated capillary length, the droplet diameter chosen for this study will not be influenced by gravitational forces. Details of the governing equations used for the simulations and mesh refinement techniques are included in the Supplementary Information (Sects. 1 and 3).

## Results and discussion

**Figure 2:**
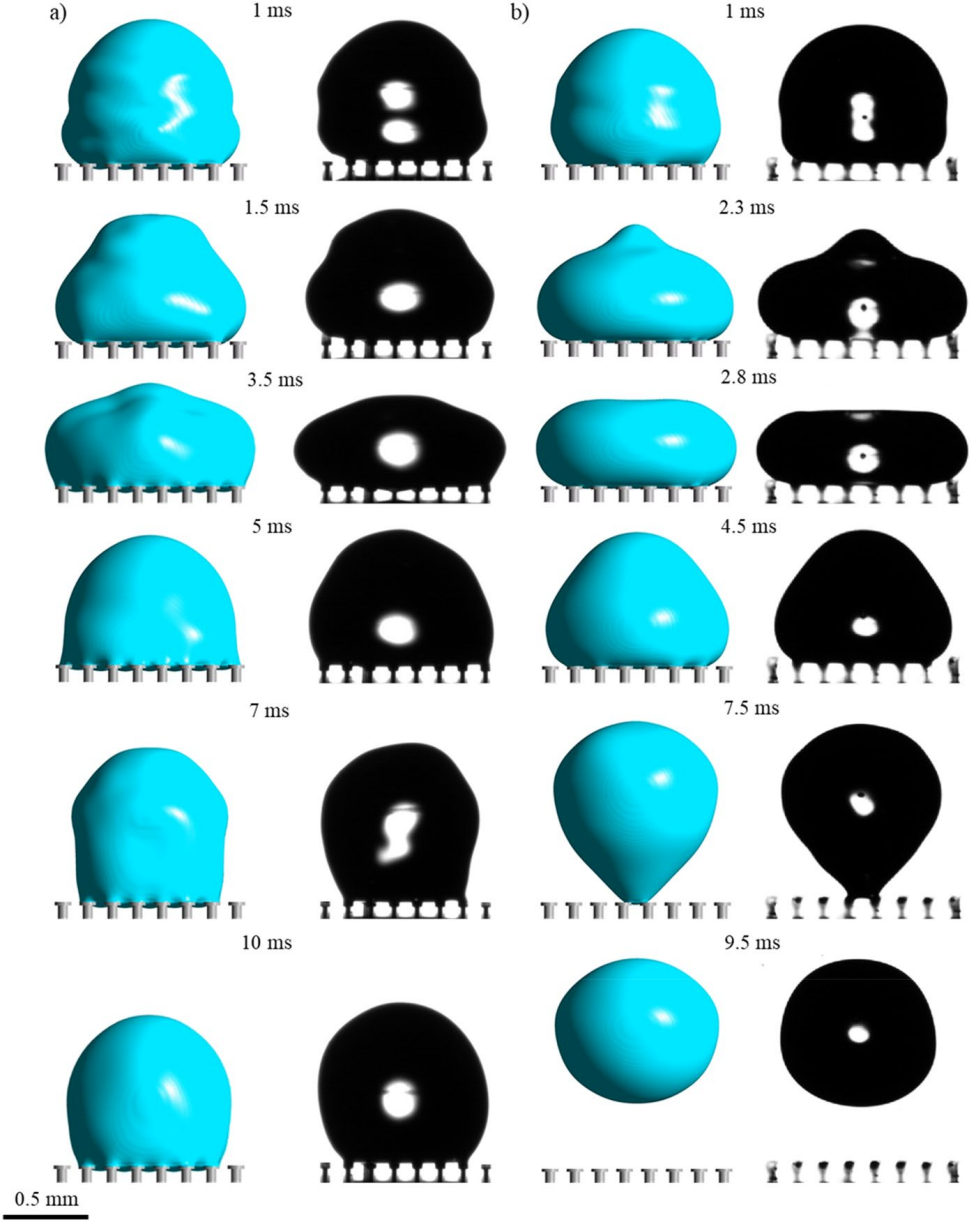
Comparison between numerical and experimental results of droplet impact at different time periods on doubly re-entrant pillars for the pitch size of 150 µm: (**a**) Pillars made of bare photoresin: V = 0.32 m/s, λ_c_ = 1.1 mm, We = 1.6; (**b**) pillars made of photoresin coated with Trichloro (1H, 1H, 2H, 2H-perfluorooctyl) silane: V = 0.33 m/s, λ_c_ = 1 mm, We = 1.5.

Figure 2 demonstrates the comparison between simulation and experimental results of droplet impact on doubly re-entrant pillars having a pitch spacing of 150 µm. The numerical results tend to agree very well with the experimental results regarding droplet spreading and repulsion at their respective timeframes. Initially, for the uncoated surface (Fig. 2a), the doubly re-entrant pillars manage to sustain the droplet due to the breakthrough pressure (*P*_c_) being greater than the total impacting pressure (*P*_d_) (1–5 ms). As the droplet attempts to recoil away from the pillars (7 ms), the droplet is instead pinned at the interface. This is primarily due to the hydrophilic nature of the uncoated photoresin, which prevents the generated capillary force from overcoming the interfacial adhesion force (Supplementary Videos S1 and S2). However, for the hydrophobized pillars (Fig. 2b), the interfacial adhesion force is greatly reduced, which allows the droplet to spread and retract on top of the pillars. The droplet eventually bounces off the surface (7.5–9.5 ms) instead of being pinned (Supplementary Videos S3 and S4).

**Figure 3:**
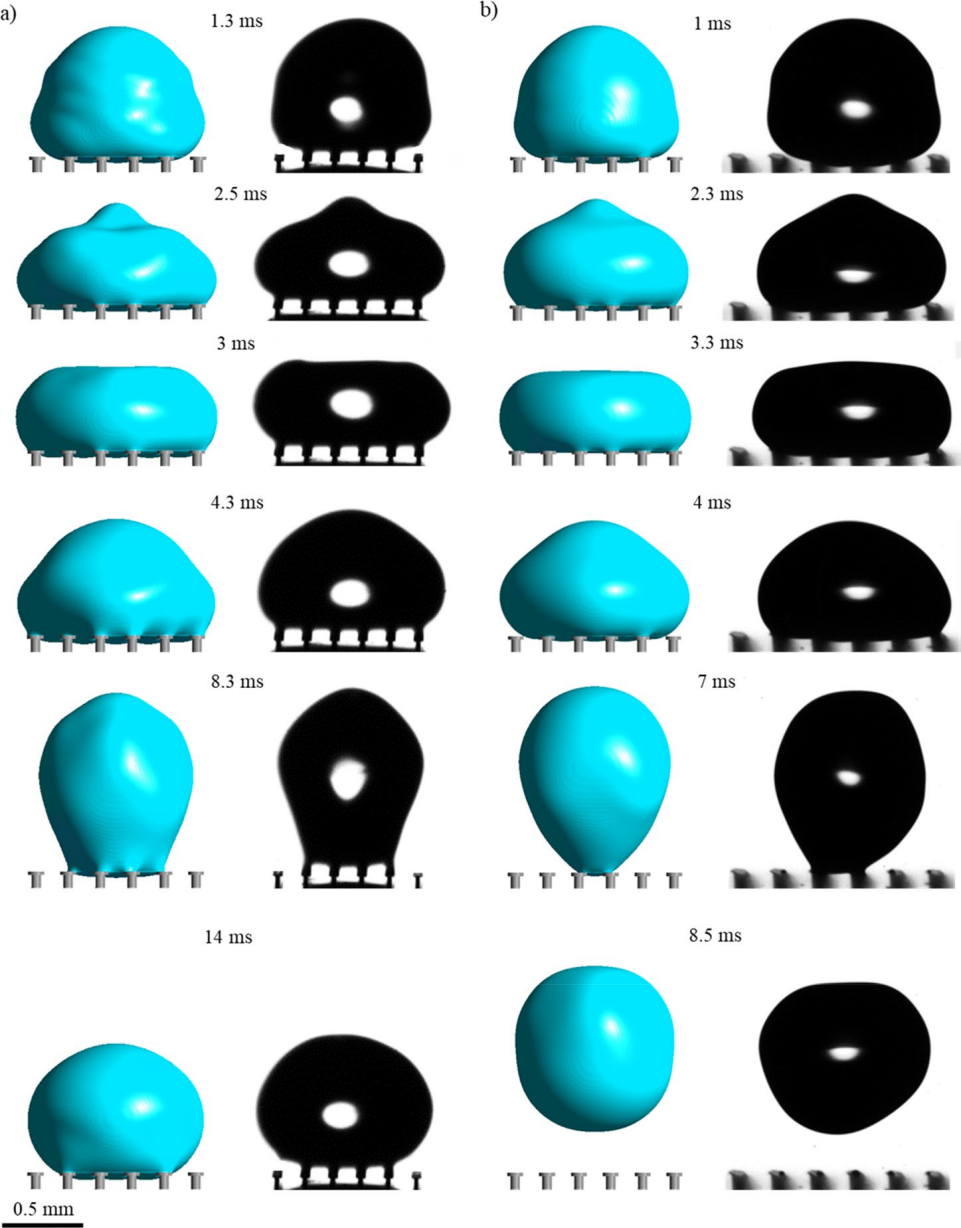
Comparison between numerical and experimental results of droplet impact on doubly re-entrant pillars at different time periods for the pitch size of 200 µm: (**a**) Pillars made of bare photoresin: V = 0.33 m/s, λ_c_** = **1.1 mm, We = 1.7; (**b**) Pillars made of photoresin coated with Trichloro (1H, 1H, 2H, 2H-perfluorooctyl) silane: V = 0.34 m/s, λ_c_** = **0.8 mm, We = 1.3.

**Figure 4:**
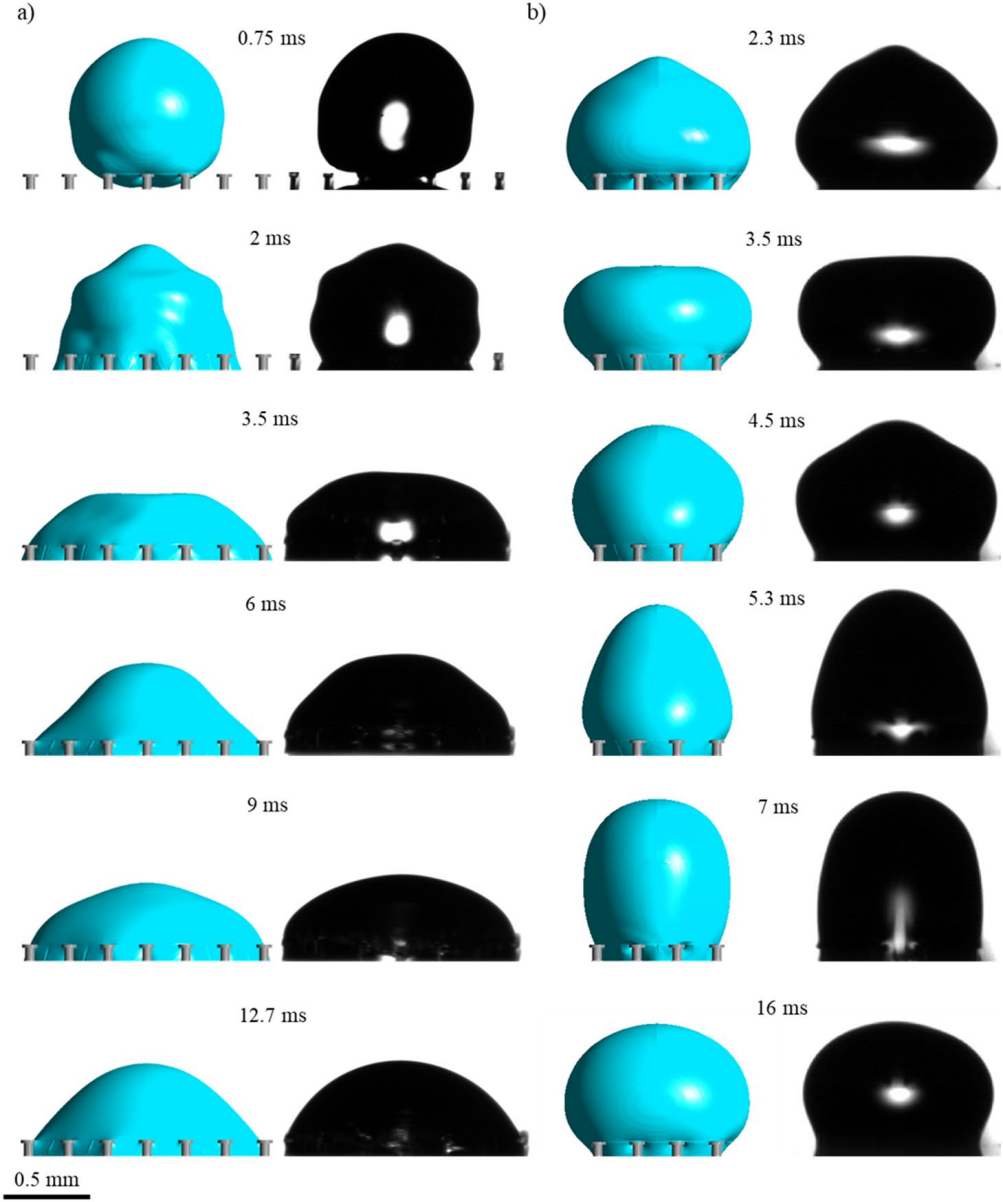
Comparison between numerical and experimental results of droplet impact on doubly re-entrant pillars at different time periods for the pitch size of 250 µm: (**a**) Pillars made of bare photoresin, V = 0.31 m/s, λ_c_ = 1.2 mm, We = 1.6; (**b**) Pillars made of photoresin coated with Trichloro (1H, 1H, 2H, 2H-perfluorooctyl) silane, V = 0.32 m/s, λ_c_ = 1.1 mm, We = 1.6.

Increasing the pitch further to 200 µm increased droplet repellency as shown in Fig. 3. This is illustrated by the reduced contact area and increased stretching of the droplet as it attempts to withdraw from the uncoated doubly re-entrant surface at 8.3 ms. Although the droplet remains pinned at the surface (seen at 14 ms), it reaches a reduced contact area, eventually, in comparison with the surface that has a 150-µm pitch (Supplementary Videos S5 and S6). From Fig. 3b, hydrophobizing the pillars shows similar results to Fig. 2b, although in this case the droplet manages to bounce off the surface at an earlier time period (7–7.8 ms) (Supplementary Videos S7 and S8). The main difference is shown by the convex meniscus at the overhang which further bulges into the cavity (seen at various timeframes) since the breakthrough pressure is reduced due to the increase in pitch size (see Supplementary Information, Sect. 5 for further details). From a kinetic point of view, increasing the pitch of the pillars reduces the interfacial adhesion force as the liquid–gas fractional area increases and the solid–liquid fractional area decreases. Nonetheless, up to a certain extent, the decline in the breakthrough pressure will inevitably reduce the droplet repellency of the surface as the amount of air pressure allowed to escape through the cavities will become dominant over the reduction in adhesion force. Therefore, a balance between the capillary and adhesion force can establish an optimal pitch value for droplet repellency. Indeed, the reduced droplet repellency performance at an even larger pitch size of 250 µm is highlighted in Fig. 4 where the droplets flood the interstices of the surfaces regardless of being hydrophobized. Figure 4a shows that the droplet spreads whilst flooding the interstices. There is a negligible lift-off (6 ms), showing that the capillary force (F_c_) is insufficient to stop the liquid from penetrating the cavities (Supplementary Videos S9 and S10). With the hydrophobized pillars in Fig. 4b, the droplet is instead confined within a smaller area of pillars where it fails to either spread or bounce off the surface (Supplementary Videos S11 and S12).

It should be noted that for the Weber numbers used for droplet impact, most of the droplets either stuck onto the pillars or flooded the cavities unless they were hydrophobized. This is attributed to the relatively large fraction of the solid area of the pillared surface (on the order of 20–50%). Here, the solid fraction (*φ*_*s*_) is referred to as the ratio of the interfacial area to the projected area of a composite surface. For doubly re-entrant pillars, this is calculated as^12,31^:

4 $$\begin{array}{c}{\varphi }_{s}=\frac{\pi {D}^{2}+4\pi D\delta }{4{S}^{2}}\end{array}$$

The surface area of the vertical overhang is included to the interfacial area which is given as $\pi D\delta$ as seen above. The derivation of the equation can be seen in the Supplementary Information (Sect. 7).

**Figure 5:**
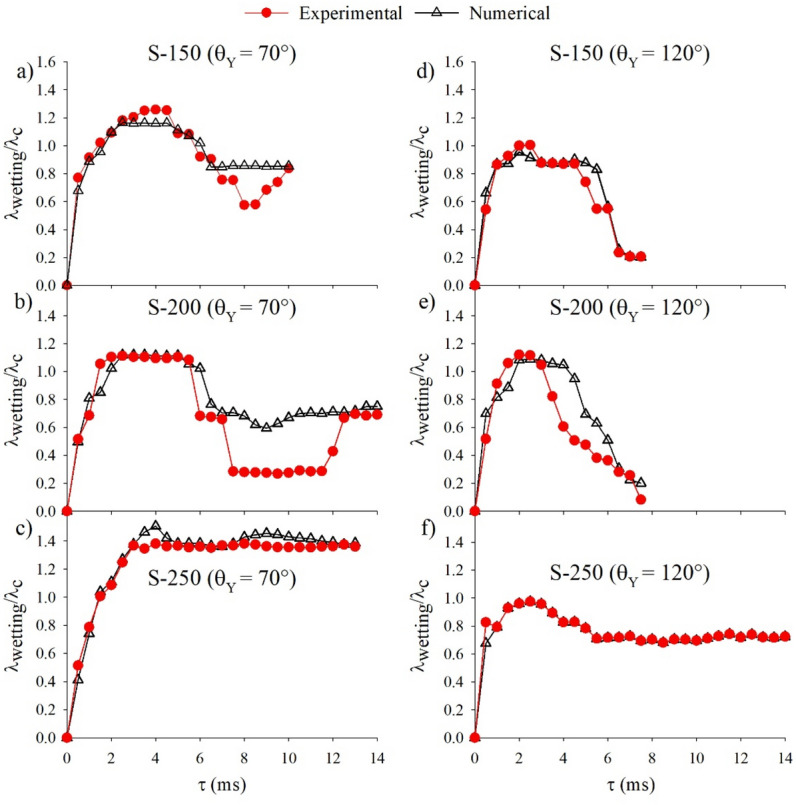
Numerical and experimental results of λ_wetting_/λ_c_ against contact time for different doubly re-entrant pitch sizes and material contact angles. (**a**) S = 150 µm, *θ*_*Y*_ = 70°; (**b**) S = 200 µm, *θ*_*Y*_ = 70°; (**c**) S = 250 µm, *θ*_*Y*_ = 70°; (**d**) S = 150 µm,* θ*_*Y*_ = 120°; (**e**) S = 200 µm, *θ*_*Y*_ = 120°; (**f**) S = 250 µm, *θ*_*Y*_ = 120°.

To quantify the comparisons, Fig. 5 displays the ratio of the maximum wetting diameter to the diameter of the droplet, λ_wetting_/λ_c_, against contact time, τ, for the processes of droplet impact corresponding to Figs. 2, 3 and 4. In general, the results further indicate good agreement between the numerical predictions and experimental results, especially for the immediate spreading regime upon impact. However, the experimentally obtained wetting diameter is shown to divert away from the numerically predicted wetting diameter in part of the retraction regime for droplets that have stayed in the Cassie state (suspended) while interacting with the pillars. This is attributed to the dissipation caused by the rupture of the thin film pinned at the top of the pillars while the contact line recedes^32^. The increase in the pitch size made this discrepancy more noticeable, because the rupture of the capillary bridge from each pillar becomes more influential when there is less solid–liquid contact area. We further note that, for the two different pitch sizes of the doubly re-entrant pillars with the same material contact angles, the maximum ratios of the Cassie-state droplets are very similar, likely because of the inertia effect that has dominated the spreading regime, especially for relatively small material contact angles^33^. Specifically, the numerically predicted maximum ratios are 1.16 and 1.12, respectively, for the untreated pillars with the pitch sizes of 150 and 200 µm, as shown in Fig. 5a and b. For the hydrophobized pillars with the pitch sizes of 150 and 200 µm, the numerically predicted ratios decrease to 0.95 and 1.09, respectively, as shown in Fig. 5d and e. By contrast, for the Wenzel-state droplets, the liquid that penetrates inside the cavities influenced the droplet behaviours by preventing the liquid from further lateral motion. Nonetheless, increasing the material contact angle has allowed the ratio to be significantly reduced for both the spreading (from 1.51 to 0.97) and retraction stages, as shown in Fig. 5c and f.

**Figure 6:**
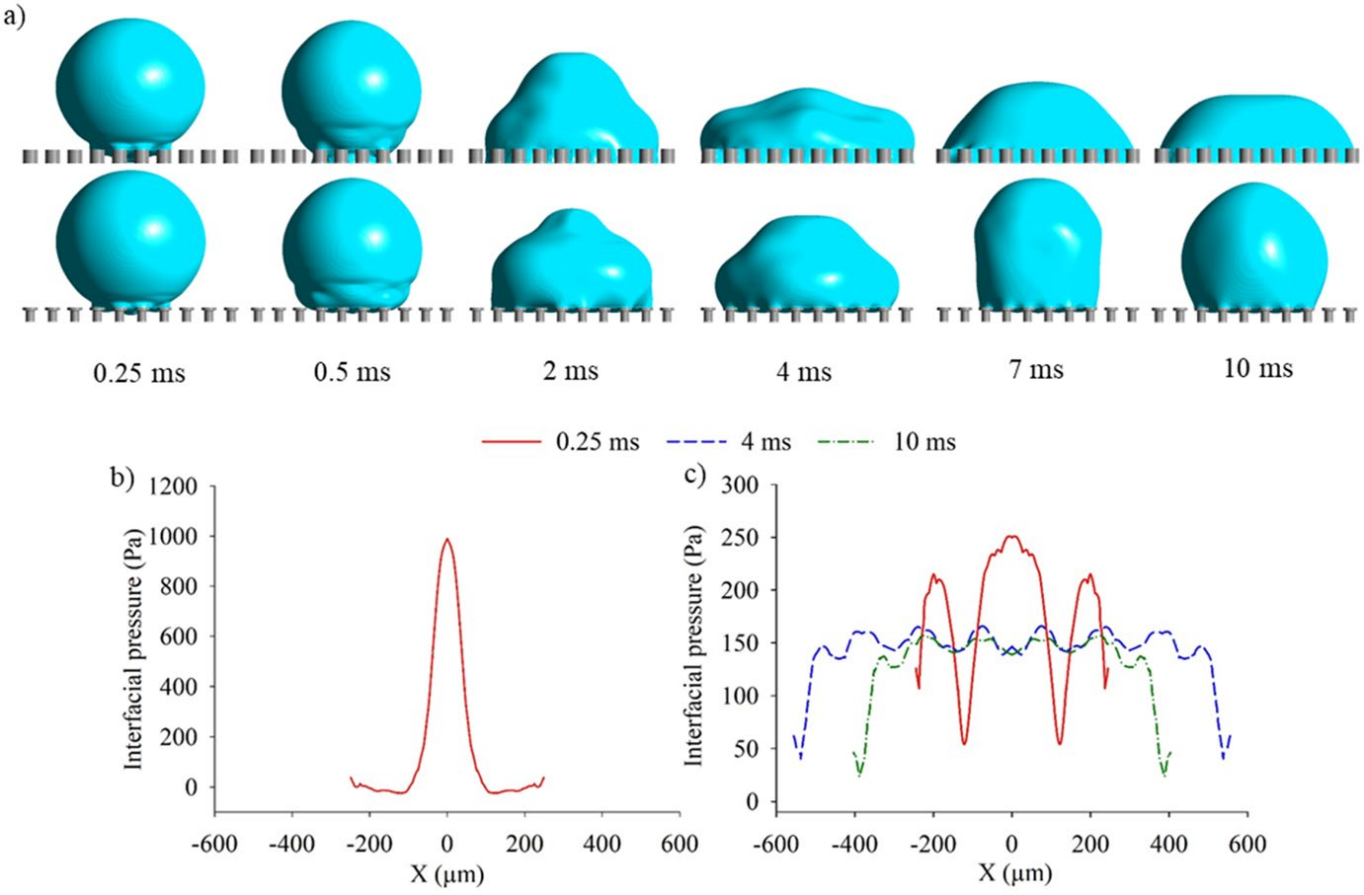
(**a**) Droplet impact behaviour at different timesteps on re-entrant and straight pillars having an intrinsic contact angle of *θ*_*Y*_ = 70°. Interfacial pressure profiles within the cavities during droplet impact for straight pillars (**b**) and re-entrant pillars (**c**), V = 0.3 m/s, λ_c_ = 1 mm, We = 1.25.

As briefly discussed, applying a horizontal and vertical overhang upon micropillars directs the surface tension force of the liquid against the direction of impact. To quantify this, the interfacial pressure profile within the pillars is depicted (Fig. 6). The interfacial pressure is the pressure experienced at the liquid–gas interface, which illustrates the pressure distribution of the air-layer confined within the cavities. Correspondingly, the penetration depth is directly related to the interfacial pressure since the penetration depth increases as the air-layer thickness is reduced. Therefore, the maximum pressure experienced beneath the droplet has been reported to be inversely proportional to the square root of the minimum air-layer thickness observed^30^. Nonetheless, it is noted that this correlation is valid for a flat surface with a two-dimensional droplet striking the surface^28,30^. Figure 6a displays the droplet impact behaviour at various timesteps for straight and re-entrant pillars having an intrinsic contact angle of 70°. Immediately upon the impact (0.25 ms), the droplet penetrates into the cavities for straight pillars, whilst on the re-entrant pillars, the droplet remains pinned at the overhang (Supplementary Videos S13 and S14). The reason for this penetration is associated with the increased interfacial pressure, as shown in Fig. 6b. Here onwards, the droplet continues to spread within the straight pillars. As the droplet has fully flooded the interstice for straight pillars after 0.25 ms, the interfacial pressure cannot be depicted. By contrast, for re-entrant pillars, the overhang structure allows the surface tension force to act against the direction of motion. This occurs when the three-phase contact line advances and suspends itself towards the bottom of the surface. Therefore, the interfacial pressure is lessened, as shown in Fig. 6c as the penetration depth is greatly reduced. The interfacial pressure profile continues to flatten and reduce as the droplet settles upon the re-entrant pillars (10 ms). Notably, the re-entrant pillars allow the droplet to recede.

**Figure 7:**
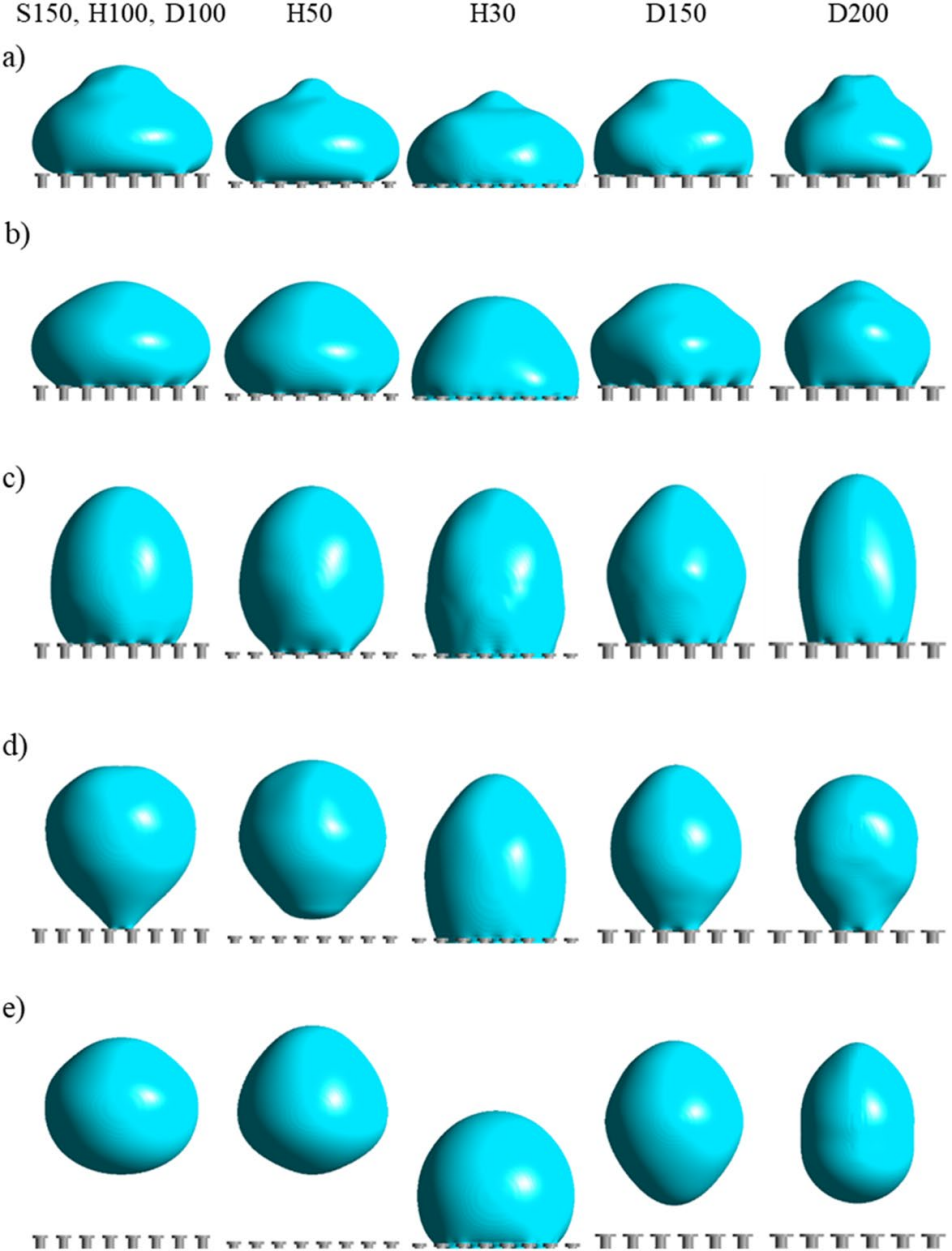
Droplet impact behaviours for various doubly re-entrant pillars with an intrinsic contact angle of *θ*_*Y*_ = 105° at (**a**) 2 ms, (**b**) 4 ms, (**c**) 6 ms, (**d**) 8 ms and (**e**) 10 ms, V = 0.3 m/s, λ_c_ = 1 mm, We = 1.25.

**Figure 8:**
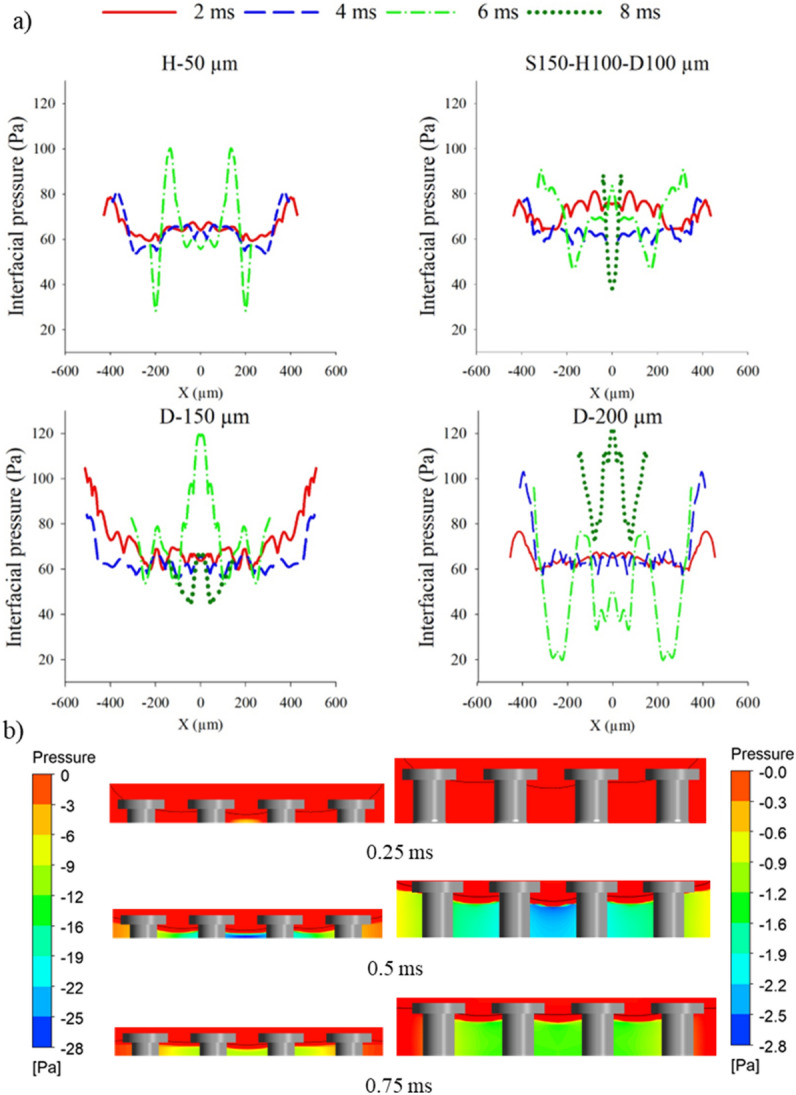
(**a**) Interfacial pressure profiles for each of the configured cases that are not flooded, (**b**) air pressure contours just after the moment of impact compared between the heights of 50 µm and 100 µm.

To determine how the dimensional parameters of the pillars affect the droplet repellence of the surface, the diameter, height, overhang length and thickness are varied. Figure 7 displays the droplet impact behaviours at various timeframes for different configurations of the doubly re-entrant pillars with the intrinsic contact angle of 105°. Figure 8a shows the interfacial pressure profiles. Increasing the diameter of the pillars from 100 to 200 µm causes the contact area of the droplet to increase before the droplet bounces (8 ms). This is primarily due to the increased adhesion force as the solid fraction of the surface becomes greater with diameter. Nevertheless, at 10 ms, the droplet manages to bounce off the surface for both the diameters of 150 and 200 µm at a lower height compared to the diameter of 100 µm (Supplementary Videos S15, S16 and S17).

Further, as indicated by the contours, decreasing the height of the pillars from 100 to 50 µm increases droplet repellency as the droplet departs off the surface by 8 ms (Supplementary Video S18). This is attributed to the increased compression of the air layer as the meniscus protrudes closer to the bottom of the cavities. Nonetheless, decreasing the pillar height towards 30 µm causes the droplet to fully wet the cavities (Supplementary Video S19). A sufficient height of the pillars allows the meniscus to be away from the bottom of the surface. Figure 8b displays this phenomenon clearly: the air pressure (absolute value) rises as the droplet protrudes closer to the bottom of the surface between 0.25 and 0.5 ms, which increases the overall resistive force against the liquid–gas interface. After 0.5 ms, the increased capillary pressure generated from air compression causes the liquid–gas interface to be repelled away from the cavities, reducing the compression, as shown from the reduced air pressure difference. It is noted that a gap between the air-layer and liquid–gas interface exists as the diffusive zone between the liquid and gas phases (see Supplementary Information, Sect. 3 for further details). The same concept is observed from expanding the pitch of the pillars up to a critical value where the meniscus is stretched further downwards. Assuming the compression is adiabatic, the ideal gas law can also be used to describe the change in pressure of the air layer as the liquid–gas interface expands further into the cavities^19,34^:

5 $$\begin{array}{c}{P}_{air}={P}_{a}\left[{\left(\frac{{v}_{i}}{{v}_{air}}\right)}^{\Gamma }-1\right]\end{array}$$

where ${P}_{air}$ is the air pressure within cavity after being compressed (Pa), ${v}_{i}$ is the initial volume of air inside the cavity (m^3^), ${v}_{air}$ is the volume of air after compression (m^3^) and $\Gamma$ is known as the specific heat ratio which is equal to 1.4 for adiabatic gas compression. Hence, from decreasing the air volume after compression, $\left(\frac{{v}_{i}}{{v}_{air}}\right)>1$, the pressure of the air layer will rise, which deflects the liquid–gas interface away.

**Figure 9:**
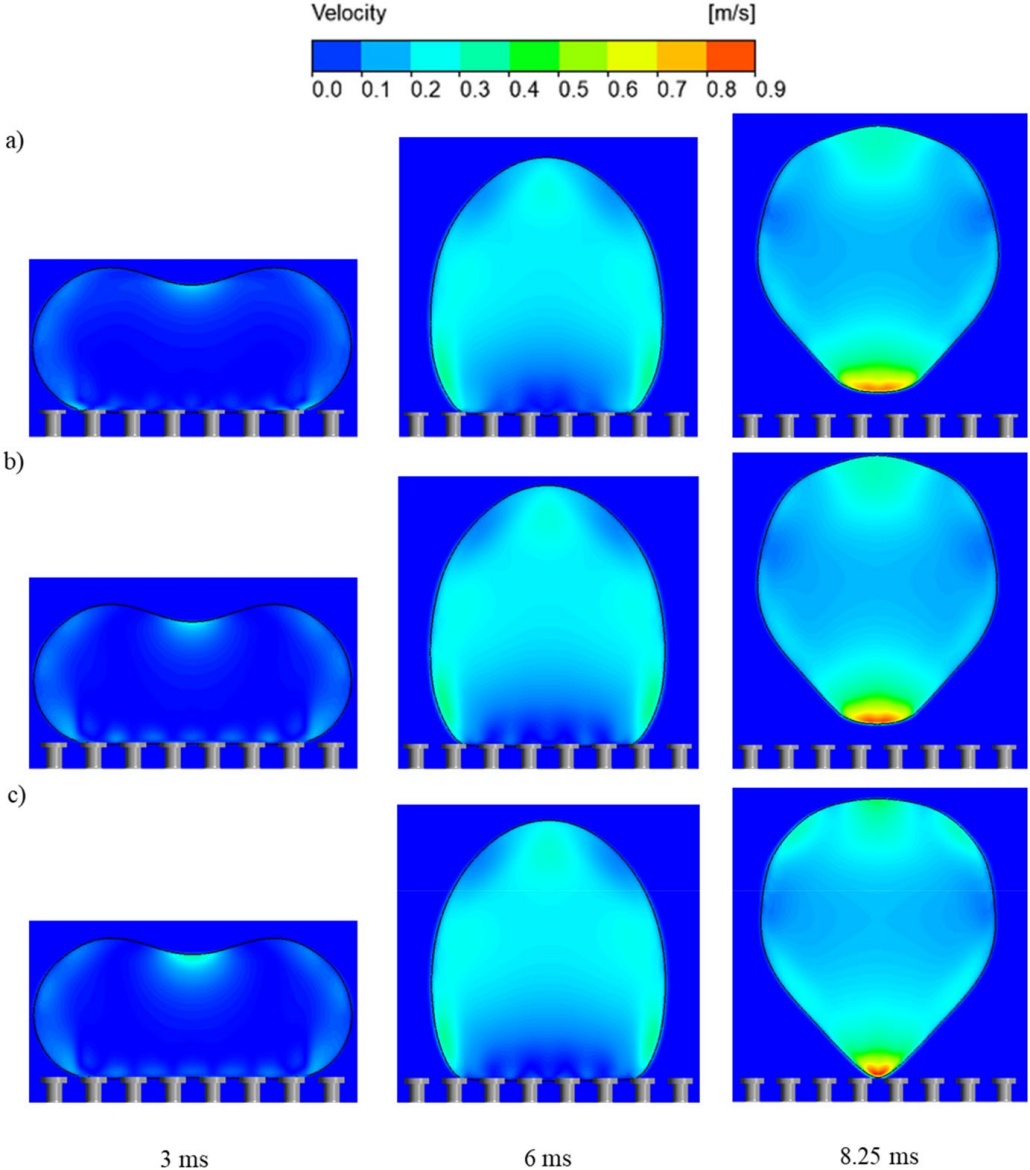
Velocity contours of droplet impacting doubly-re-entrant pillars at different time periods with different overhang lengths. (**a**) δ = 5 µm, (**b**) δ = 10 µm, (**c**) δ = 15 µm.

To explore the effects of the overhang length (δ) on the droplet repellency of the surface, Fig. 9 represents the velocity contours of the droplet at different time periods for the configured overhang lengths. Initially, as the droplet begins to recoil back (3 ms), the velocity is shown to be larger at the apex of the droplet for larger overhang lengths. At 8.25 ms, the droplet is still in contact with the pillars that have an overhang length of δ = 15 µm. By contrast, for the smaller overhang lengths of 10 µm and 5 µm, the droplets have already departed off the pillars (Supplementary Videos S20 and S21). This is due to the increased projection of the meniscus at the centre of impact, which delays the onset of repellency. As shown in Fig. 1f, for doubly re-entrant pillars, the liquid meniscus can be pinned at the bottom of the vertical overhang. Increasing the length of the overhang further will not only cause the meniscus to approach the bottom of the cavities, but also require more time for the receding liquid to detach from the pillars. Therefore, while the presence of the vertical overhang is essential for the pillars to suspend the wetting liquid, the length of the vertical overhang should be minimised to keep the solid fraction that can be contacted by the wetting liquid at bay^12^.

**Figure 10:**
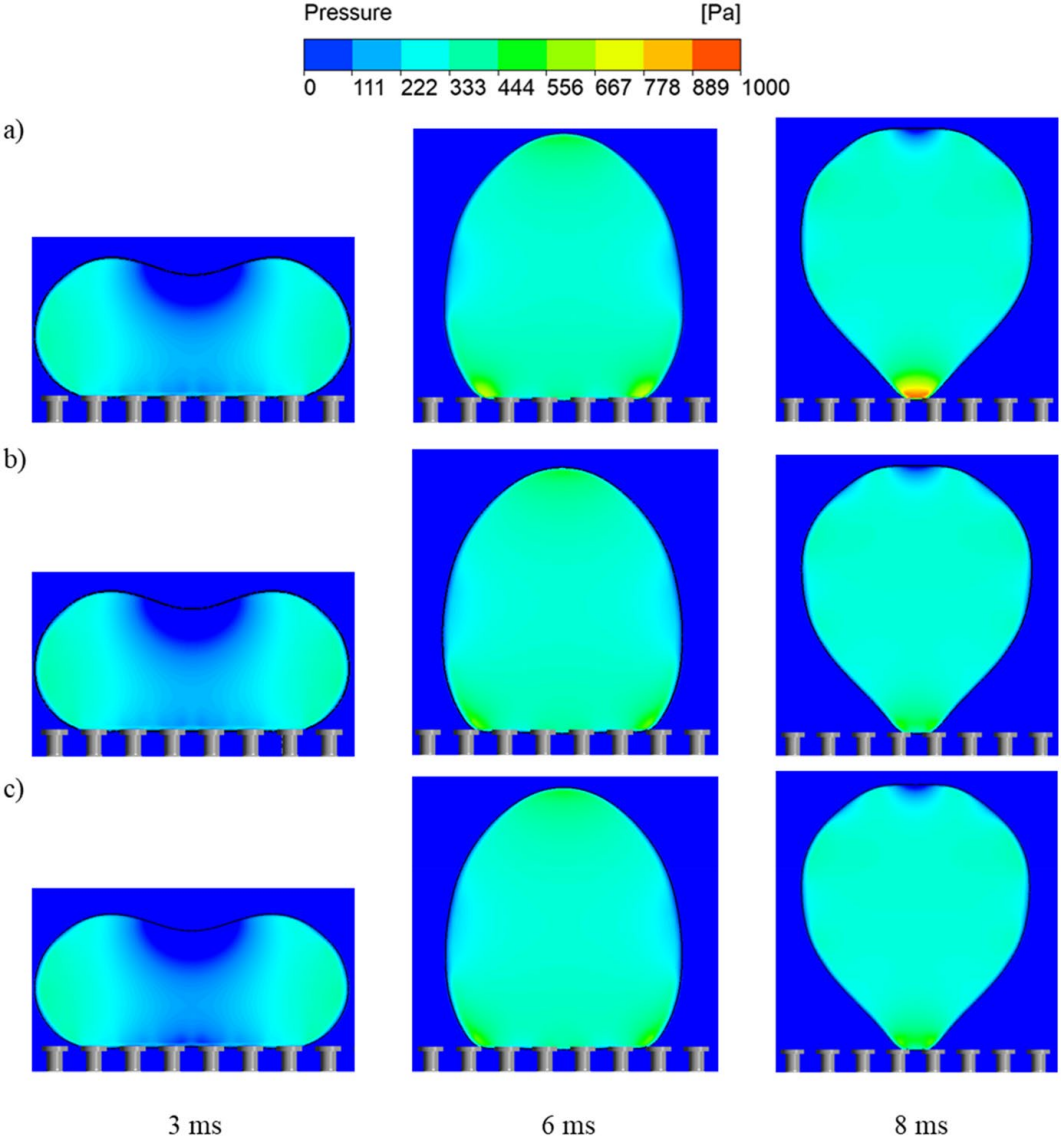
Pressure contours of impacting droplets on doubly re-entrant pillars at multiple time steps for various overhang thicknesses: (**a**) t_o_ = 5 µm, (**b**) t_o_ = 10 µm, (**c**) t_o_ = 15 µm.

Figure 10 displays the pressure contours during droplet impact for various timesteps and overhang thicknesses. From decreasing the thickness (t_o_) from 15 to 5 µm, the base of the droplet experiences an increase in capillary pressure that is demonstrated at 8 ms due to the reduction in the wetting diameter (Supplementary Videos S22 and S23). The additional pressure experienced against the droplet at lower overhang thickness can be predicted (Supplementary Information, Sect. 5, Eq. S18). Reducing the overhang thickness causes the diameter of the circular contact line at the bottom of the vertical overhang (*D′*) to become nearly equal to diameter of the pillars (*D′* ≈ *D*). Nevertheless, as indicated from the results, varying the thickness between 5 and 15 µm provides only a slight change in droplet repellency.

**Figure 11:**
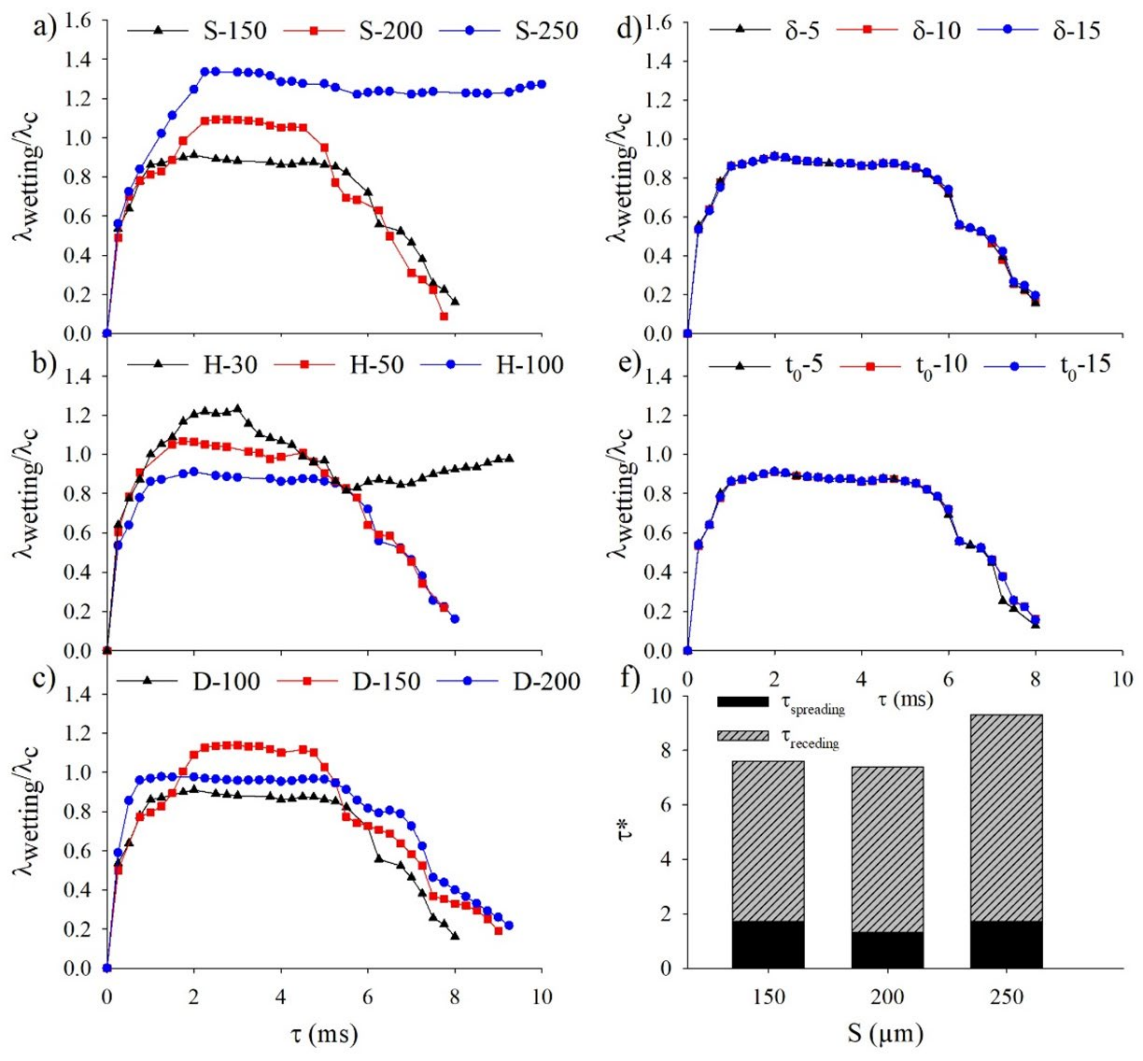
λ_wetting_/λ_c_ against contact time for each of the dimensional parameters varied: (**a**) the pitch spacing (S); (**b**) pillar height (H); (**c**) pillar diameter (D); (**d**) length of the vertical overhang (δ); and (**e**) the thickness of the vertical overhang (t_o_). *θ*_*Y*_ = 105°. (**f**) dimensionless contact time (τ*) against pitch spacing (S) chart highlighting the time taken for the droplet to spread (τ_spreading_) and recede (τ_receding_) on each of the configured surfaces.

Figure 11 demonstrates the ratio of the maximum wetting diameter to the diameter of the droplet, λ_wetting_/λ_c_, against contact time for the doubly re-entrant pillar dimensions with the material contact angle of 105° varied in the simulations. From Fig. 11a, increasing the pitch size from 150 to 200 µm results in an initial increase in droplet spreading. However, the droplet manages to detach from the 200 µm surface at an earlier contact time (Supplementary Video S24). This is primarily due to the reduction in the solid fractional area. Further increasing the pitch towards 250 µm causes λ_wetting_/λ_c_ to dramatically increase and remain roughly within the same range (around 1.3) throughout the contact time (between 2 to 10 ms). This is due to the fact that the droplet does not bounce off the surface. It, instead, remains on the surface (Supplementary Video S25). It should be noted that, when comparing Fig. 11a to Fig. 5 for different intrinsic contact angles and pitch sizes, increasing the intrinsic contact angle from 70° to 105° reduces λ_wetting_/λ_c_ as the droplet rebounds instead of being pinned for pitch sizes of 150 and 200 µm. When the material contact angle is increased from 105° to 120°, λ_wetting_/λ_c_ remains approximately the same throughout the spreading period. However, it takes longer for the droplet to recede and rebound at 105° compared to 120°. As mentioned previously, increasing the material contact angle reduces the interfacial adhesion force, which then reduces λ_wetting_/λ_c_ for a given pitch size^25,35^. For the pitch size of 250 µm, the magnitude of λ_wetting_/λ_c_ at 105° is also placed between 120° and 70° (Fig. 5).

Figure 11b shows that reducing the height of the pillars from 100 to 30 µm also causes λ_wetting_/λ_c_ to gradually increase. It should be noted that, even for the smallest pillar height of 30 µm that allows the droplet to spread, λ_wetting_/λ_c_ still manages to reach a value of around 0.8 at roughly 6 ms. This indicates that the spreading of the droplet is less triggered by the reduced height than by the increased pitch of the doubly re-entrant pillars. Figure 11c illustrates the values of λ_wetting_/λ_c_ for pillar diameters between 100 and 200 µm. Increasing the diameter from 100 to 150 µm causes λ_wetting_/λ_c_ to increase, because of the increased solid fraction. When increasing the diameter from 150 and 200 µm, however, λ_wetting_/λ_c_ reduces slightly by 0.2 between 2 to 5 ms. As the droplet is in contact with a smaller number of pillars when increasing the diameter to 200 µm (as seen from Fig. 7), the contact line is pinned at a smaller distance (Supplementary Information, Sect. 8). In spite of this, the droplet still sits longer on the doubly re-entrant pillars with a diameter of 200 µm than on those with a diameter of 150 µm, where the droplet starts receding after 5 ms, and eventually, bounces off the surface prior to that of the larger pillar diameter of 200 µm. In contrast, the length and thickness of the vertical overhang have only negligible effect on λ_wetting_/λ_c_ as shown in Fig. 11d and e.

Since the pitch of the doubly re-entrant pillars has exhibited the most significant effect on λ_wetting_/λ_c_ among the factors, Fig. 11f further scrutinises the correlation between the dimensionless contact time, τ*, and the pitch size. τ* is defined as the ratio of the contact time to the inertial-capillary contact time (${\tau }^{*}= \frac{\tau }{{\tau }_{o}}$). The inertial-capillary contact time is specified as the contact time for a droplet impacting on a flat superhydrophobic surface which is scaled as^36,37^:

6 $$\begin{array}{c}{\tau }_{o}=\sqrt{\frac{{\rho }_{l}{R}^{3}}{{\gamma }_{lg}}}\end{array}$$

For *We* > 1, the inertial-capillary contact time is independent of the impact velocity^37^. For a water droplet with a diameter of 1 mm, the inertial-capillary contact time is equal to 1.32 ms. From the chart, increasing the pitch size from 150 to 200 µm gradually decreases the overall contact time. Specifically, the time taken for the droplet to spread (τ_spreading_) slightly decreases. Further increasing the pitch towards 250 µm displays a substantial increase in the contact time as the liquid fully penetrates the cavities of the pillars and floods the surface. As a consequence, the time taken for the droplet to recede is substantially longer since the liquid is adhered to the bottom of the pillars.

## Conclusion

Numerical simulations and experimental studies of droplet impact against doubly re-entrant pillars have been conducted to probe the underlying physical phenomena. Good agreement concerning the droplet behaviours has been found between the numerical and experimental results. This is demonstrated through both the comparison of the contour images and the quantitative analysis of the ratio of the maximum wetting diameter to the diameter of the droplet, λ_wetting_/λ_c_, against contact time for the various processes of droplet impact. Therefore, direct numerical simulations based on a Volume-of-Fluid method can be used to further support our understanding of droplet behaviours resulting from impacting complex surface structures. Specifically, the pitch, height, diameter, overhang length and thickness of the doubly re-entrant pillars have been varied to illustrate their roles in governing droplet repellency. Among these dimensional parameters, the pitch of the doubly re-entrant pillars has exhibited the most significant effect on the evolution of λ_wetting_/λ_c_. Additionally, the velocity and pressure contours have been displayed to identify the region of significance. This study provides an improved understanding of how adding the horizontal and vertical overhang to straight pillars enables droplet repellency for re-entrant and doubly re-entrant pillars.

### Supplementary Information

Supplementary Information.

Supplementary Video 1.

Supplementary Video 2.

Supplementary Video 3.

Supplementary Video 4.

Supplementary Video 5.

Supplementary Video 6.

Supplementary Video 7.

Supplementary Video 8.

Supplementary Video 9.

Supplementary Video 10.

Supplementary Video 11.

Supplementary Video 12.

Supplementary Video 13.

Supplementary Video 14.

Supplementary Video 15.

Supplementary Video 16.

Supplementary Video 17.

Supplementary Video 18.

Supplementary Video 19.

Supplementary Video 20.

Supplementary Video 21.

Supplementary Video 22.

Supplementary Video 23.

Supplementary Video 24.

Supplementary Video 25.

## Data Availability

All data generated or analysed during this study are included in this published article (and its Supplementary Information file).
