# Supplementary material for: Droplet impact on doubly re-entrant structures
Source: Sci Rep. 2024 Feb 1;14:2700. doi: 10.1038/s41598-024-52951-2 (PMC10834531; doi:10.1038/s41598-024-52951-2)
Supplement: Supplementary file 1 — Supplementary Information. [file 41598_2024_52951_MOESM1_ESM.docx]

**Droplet impact on doubly re-entrant structures**

*Navdeep Sangeet Singh^1^*, *Thanaphun Jitniyom^1^, Miguel Navarro-Cía^1,2^,* *Nan Gao^1, *^*

*^1^School of Engineering, University of Birmingham, Birmingham, B15 2TT, United Kingdom*

*^2^School of Physics and Astronomy, University of Birmingham, Birmingham, B15 2TT, United Kingdom*

** Correspondence concerning this manuscript should address N.G. at* [*n.gao@bham.ac.uk.*](mailto:n.gao@bham.ac.uk.)

**1. Cases run for simulation and mesh refinement**

**Table S1** displays the cases run for each of the configurated pillars, illustrating their dimensions and intrinsic contact angles settings. Additionally, **Figure S1** highlights the key dimensions configured on each pillar type.

Table S1. Dimensions and intrinsic contact angles of the pillars used for the simulations.

a)

b)

c)


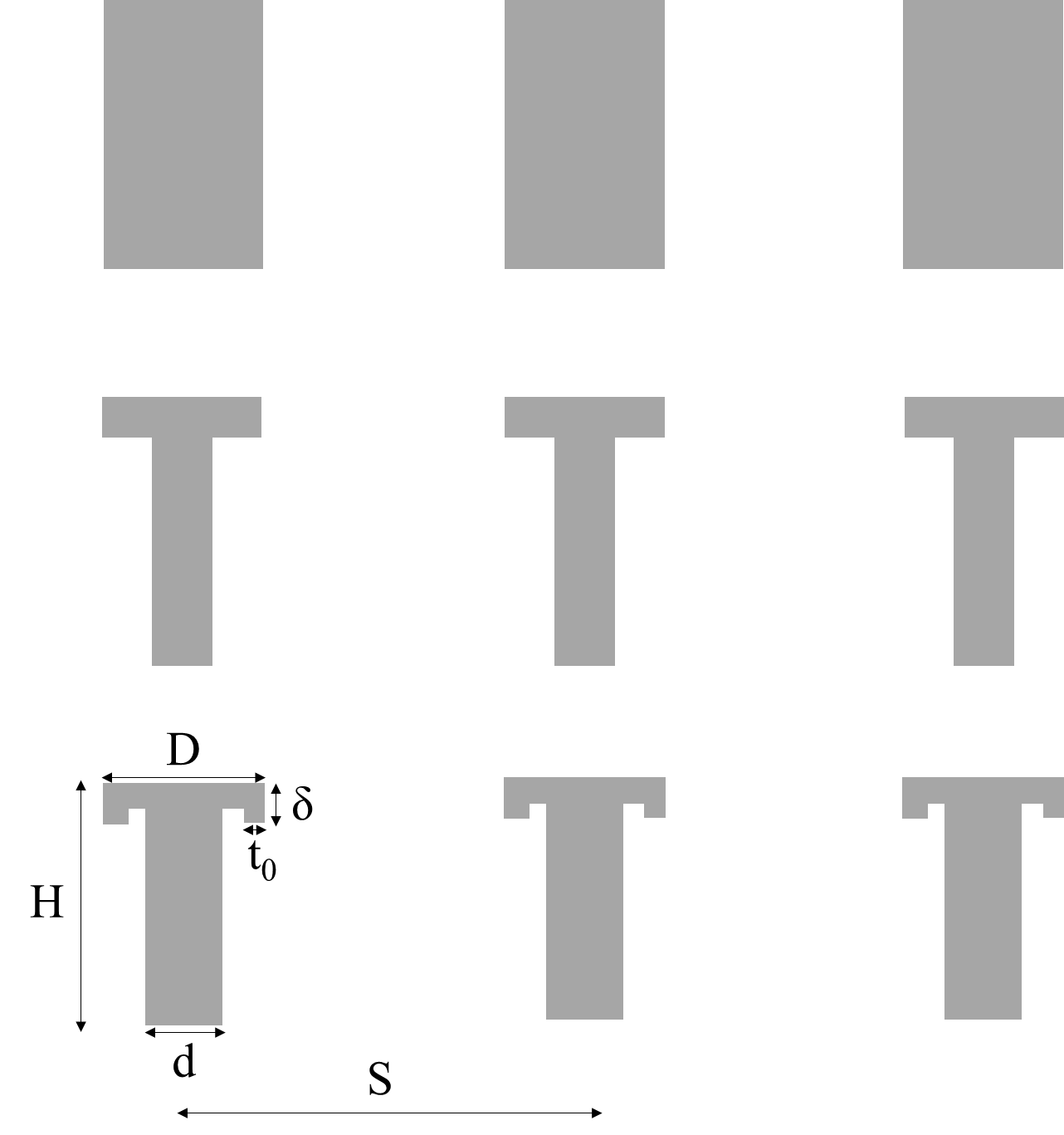


Figure S1. Key dimensions for a) straight circular, b) re-entrant and c) doubly re-entrant pillars.

**Figure S2** illustrates the hexahedral elements that were used to mesh the fluid domain and pillars. To adequately interpolate the liquid-gas interface of the droplet during impaction and repulsion, mesh adaption was applied at the bottom and top sections of the fluid, with a level one octree mesh refinement where the mesh elements were split into quarters. According to the mesh sensitivity study by Hu *et al.*, the thickness of the air layer within the capillaries was able to be appropriately captured for element numbers greater than or equal to one million [14]. Therefore, the number of mesh elements in all our cases was ensured to be more than one million.


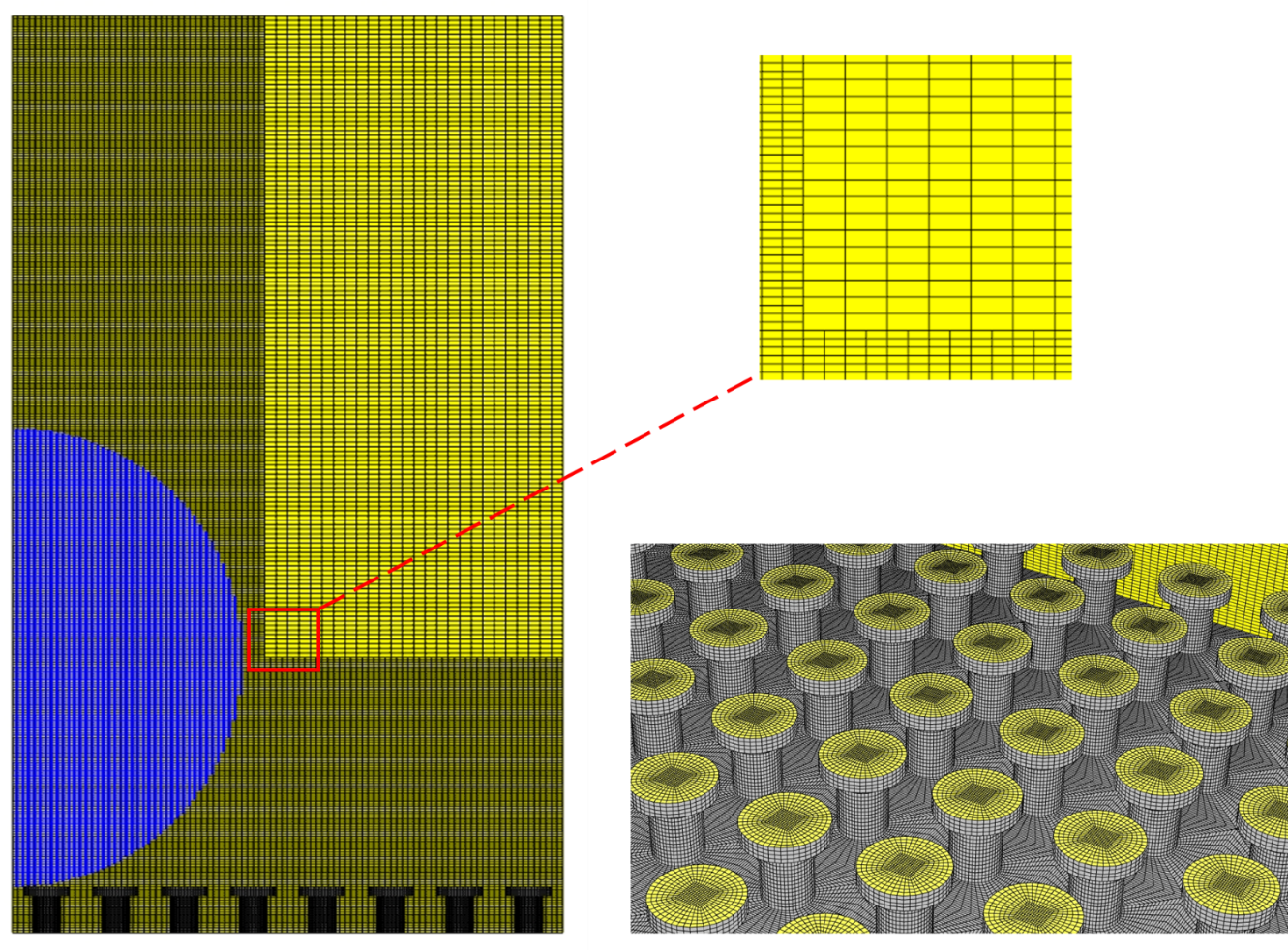


a)

b)

Figure S2. a) Mesh topology of fluid domain having a level one octree mesh adaption which is applied within the vicinity of the droplet impact and repulsion area as elucidated from the darker regions, b) Structured mesh refinement applied upon the doubly re-entrant pillar arrays.

**2. Contact angle measurements**

The static and dynamic contact angles on each of our plain and structured surfaces were measured using a goniometer (Data-Physics OCA 15) at room temperature. A sessile drop (6 𝜇L) method was used to measure the static contact angles. For the dynamic contact angles, the volume of the sessile drop was increased and decreased to measure the advancing and receding contact angles, respectively, whilst performing drop shape analysis. For example, the minimum dosing rate used to add and withdraw the liquid was 0.16 µL/s. The measurements were repeated 3 times for each surface type to determine the average contact angles and the standard deviations. Table S2 presents the contact angle measurements for each surface type alongside the calculated contact angle hysteresis.

Table S2. Static and dynamic contact angle measurements of water on the fabricated doubly re-entrant pillars with and without the (trichloro (1H,1H, 2H, 2H-perfluorooctyl) silane (PFOCTS) coating. *θ_c_* is the apparent contact angle (°), *θ_a_* is the advancing contact angle (°), *θ_r_* is the receding contact angle (°), and *Δθ* is the contact angle hysteresis (*θ_a_ – θ_r_)*.

| **Surface type** | ***θ_c_* (°)** | ***θ_a_* (°)** | ***θ_r_* (°)** | ***Δθ* (°)** |
| --- | --- | --- | --- | --- |
| IP-S | 70.7 ± 1.0 | 81.8 ± 2.0 | 37.2 ± 8.7 | 44.6 ± 8.9 |
| IP-S + PFOCTS | 112.9 ± 4.9 | 113.4 ± 2.6 | 73.3 ± 4.3 | 40.1 ± 5.0 |
| IP-S (S-150 µm) | 139.3 ± 0.3 | 152.5 ± 3.2 | 132.3 ± 3.4 | 20.2 ± 4.7 |
| IP-S (S-200 µm) | 141.0 ± 0.4 | 151.0 ± 3.7 | 135.0 ± 4.1 | 16.0 ± 5.5 |
| IP-S (S-250 µm) | 142.9 ± 0.2 | 153.7 ± 2.5 | 137.7 ± 3.1 | 16.0 ± 4.0 |
| IP-S + PFOCTS (S-150 µm) | 140.8 ± 0.3 | 154.5 ± 4.4 | 136.0 ± 4.0 | 18.5 ± 6.0 |
| IP-S + PFOCTS (S-200 µm) | 142.4 ± 0.8 | 153.4 ± 3.3 | 139.4 ± 2.7 | 14.1 ± 4.2 |
| IP-S + PFOCTS (S-250 µm) | 143.9 ± 0.4 | 153.5 ± 2.1 | 140.4 ± 1.6 | 13.1 ± 2.7 |

**3. Numerical methods**

To capture the liquid-gas interface of the droplet, the Volume of Fluid method (VOF) is used to prescribe the volume fraction of each phase which is determined from the mass conservation equation:

$$\begin{aligned} \frac{1}{\rho_{q}}\left( \frac{\partial}{\partial t}\left( \alpha_{q}\rho_{q} \right)+\nabla\cdot\left( \alpha_{q}\rho_{q}\vec{v_{q}} \right) \right)=0 \#\left( S SEQ ( \backslash* ARABIC 1 \right) \end{aligned}$$

where *q* either refers to the liquid (l) or gas (g) phase. Furthermore, the momentum conservation equation is introduced to calculate the velocity and pressure of each of the phases:

$$\begin{aligned} \frac{\partial}{\partial t}\left( \rho\vec{v} \right)+\nabla\cdot\left( \rho\vec{v}\vec{v} \right)=-\nabla p+\nabla\cdot\left[ \mu\left( \nabla\vec{v}+\nabla\vec{v}^{T} \right) \right]+\rho\vec{g}+\vec{F_{vol}} \#\left( S SEQ ( \backslash* ARABIC 2 \right) \end{aligned}$$

Here, $\vec{F_{vol}}$ stands for the source term which is used to allocate the interfacial surface tension force at the liquid-gas interface. Additionally, the momentum equation specified here is resolved within the mixture phase (i.e. where a cell has a volume fraction between 0 and 1). As a result, the density and dynamic viscosity imposed are computed as follows:

$$\begin{aligned} \rho=\alpha_{l}\rho_{l}+\left( 1-\alpha_{l} \right)\rho_{g} \#\left( S SEQ ( \backslash* ARABIC 3 \right) \end{aligned}$$

$$\begin{aligned} \mu=\alpha_{l}\mu_{l}+\left( 1-\alpha_{l} \right)\mu_{g}\#\left( S SEQ ( \backslash* ARABIC 4 \right) \end{aligned}$$

Notably, $\left( 1-\alpha_{l} \right)$ can be re-written as $\alpha_{g}$, as the calculated volume fraction is based on the secondary phase involved which is the liquid phase ($\alpha_{l}$). To determine the surface tension force, the continuum surface tension force model (CSF) (established by Brackbill *et al.*) is implemented by calculating the interfacial pressure gradient between the two phases [1]:

$$\begin{aligned} F_{vol}=\gamma_{lg}\frac{\rho K\nabla\alpha_{l}}{\frac{1}{2}\left( \rho_{g}+\rho_{l} \right)} \#\left( S SEQ ( \backslash* ARABIC 5 \right) \end{aligned}$$

$K$, here, represents the interfacial surface curvature that is defined by taking the divergence of the unit normal vector [1]:

$$\begin{aligned} K=\nabla\cdot\hat{n}\#\left( S SEQ ( \backslash* ARABIC 6 \right) \end{aligned}$$

$\hat{n}$ is given as the unit normal vector. The surface curvature is determined by the value of the volume fraction gradient normal to the surface. Thus, the surface normal can be defined as the volume fraction gradient of the secondary phase:

$$\begin{aligned} n= \nabla\alpha_{l}\#\left( S SEQ ( \backslash* ARABIC 7 \right) \end{aligned}$$

Hence, the unit normal vector is given as:

$$\begin{aligned} \hat{n}=\frac{n}{\left| n \right|}\#\left( S SEQ ( \backslash* ARABIC 8 \right) \end{aligned}$$

To prescribe the effect of the intrinsic contact angle, a wall adhesion model is introduced which calculates the surface adhesion force as a function of the contact angle (*θ_Y_*) at the wall interface. By altering the intrinsic contact angle, the unit normal vector at the wall interface is revealed as:

$$\begin{aligned} \hat{n}=\hat{n}_{w}\cos\theta_{Y}+\hat{t}_{w}\sin\theta_{Y}\#\left( S SEQ ( \backslash* ARABIC 9 \right) \end{aligned}$$

$\hat{n}_{w}$ and $\hat{t}_{w}$ are known as the normal and tangential unit vectors at the wall. Furthermore, the pressure and velocity correction gradients are introduced and coupled in the simulation to prevent divergence in the continuity equations. This is done using the PISO (Pressure-Implicit with Splitting of Operators) method which repeats the calculations (or iterations) until the mass and momentum equations contain a normalized residual value of ≤ 10^-3^. Likewise, the finite volume method is used to solve the mass and momentum equations as it is inherent to ANSYS Fluent. A second-order upwind scheme is implemented alongside to discretise the continuity equations which enhances the accuracy of the solution. Correspondingly, the compressive scheme is used to interpolate the advection between the liquid and gas [2]. Using sharper discretisation schemes may result in spurious oscillations at the liquid-gas interface. These instabilities typically proliferate throughout the flow (for example, during droplet spreading and receding) and produce fictitious flow instabilities [3]. To avoid this, the compressive method uses a slope limiter value which can be varied between 0 and 2 depending upon the resolution needed. Therefore, the volume fraction value on a face element is adjusted as shown below [2]:

$$\begin{aligned} \alpha_{f}=\alpha_{d}+\beta\nabla\alpha_{d}\cdot d\vec{r} \#\left( S SEQ ( \backslash* ARABIC 10 \right) \end{aligned}$$

$\alpha_{f}$ is given as the face VOF value, $\alpha_{d}$ is the donor cell VOF value, $\beta$ is the slope limiter value, $\nabla\alpha_{d}$ is known as the donor cell VOF gradient value and $d\vec{r}$ is the cell to face distance. To obtain a sufficiently sharp interface, a slope limiter value of 2 is chosen. As the simulation is transient (i.e. time-dependent), the unsteady-state variable in the continuity equations is solved implicitly using the bounded second-order implicit method. Combining this with the compressive scheme produces a stable robust liquid-gas interface, as the diffusive zone near the interface is decreased (i.e. the boundary where 0 < *α_q_* < 1 lies). As noted formerly, this will further lessen any conjured flow fluctuations.

It should be noted that a droplet will induce a shock pressure that is generated upon impact. Due to the sudden rise in pressure, a capillary wave is generated at the liquid-solid interface which propagates against the direction of impact [4]. To appropriately apprehend the capillary wave without creating flow instability, the hydrodynamics displayed within a single mesh element must be captured as the capillary wave is transmitted across each element. Therefore, the time-step given must be varied as a function of the capillary waves’ velocity. The Courant number (also known as the Courant–Friedrichs–Lewy condition) is used to meet this requirement which is illustrated as [5]:

$$\begin{aligned} Co= \frac{a\Delta t}{\Delta x}\#\left( S SEQ ( \backslash* ARABIC 11 \right) \end{aligned}$$

*a* is the wave velocity (m/s), with $\Delta t$ being the timestep (s) and $\Delta x$ the size of the elements (m). As the Courant number reaches more than one, the velocity field will start to diverge as the capillary wave will travel through more than one element at a time, which, in turn, will misrepresent the flow behaviour. To avoid this phenomenon, a Courant number of 0.2 was set [6] where the time-step routinely alters its value to satisfy this condition by utilizing an adaptive time-stepping method.

**4. Fabrication of doubly re-entrant pillars**

The configured doubly re-entrant pillars were designed using Autodesk 360 fusion and saved as a stereolithography file (STL), which was imported into Describe (Version 2.7, Nanoscribe GmbH, Germany) to prepare for laser lithography via a Nanoscribe Photonic Professional GT+ 3D printer. For all 3D printing processes of the doubly re-entrant pillars, a commercially available liquid photoresin (IP-S, Nanoscribe GmbH, Germany) was utilised. The photoresin was first applied onto indium tin oxide coated glass substrates (ITO glass). A 25x/NA 0.8 objective lens (Carl Zeiss AG, Germany) was then used to construct the pillars from the photoresin. The printing process was carried out with a piezo setting time of 10 ms, galvo acceleration of 10 V/s^2^, stage velocity of 200 µm/s, laser power of 100 mW, and scan speed of 100 mm/s. The slicing and hatching distances were 1 μm and 0.5 μm, respectively, when the solid printing mode was used with no contour count and a base slice count of 2.

Following the printing process, the structures were developed in propylene glycol methyl ether acetate (PGMEA) for 20 minutes, followed by development in isopropyl alcohol (IPA) for 5 minutes. After the completion of the development process, the pillars were then air-dried at room temperature. By using scanning electron microscopy (SEM) imaging, we visually examined the 3D printed objects for their consistency in terms of their dimensions and overall structure. To improve the quality of the images, the structures were coated with gold before being observed using SEM.

To hydrophobize the surfaces, the fabricated doubly re-entrant pillars were placed in a vacuum desiccator at room temperature with 30 µL of (trichloro (1H,1H, 2H, 2H-perfluorooctyl) silane (PFOCTS) (97%, Sigma Aldrich) placed on a glass slide next to the surfaces for 90 minutes to allow chemical vapour deposition to occur. Once coated, the surfaces were removed from the vacuum desiccator and left overnight for 20 hours. This further increased the intrinsic contact angle to 112.9 ± 4.9° from 70.7 ± 1.0, as shown in Table S2. For simplicity, the intrinsic contact angle was rounded up to 120° to represent the doubly re-entrant pillars that had been hydrophobized.

**5. How micro-pillars repel impacting droplets**

If the total impacting pressure is greater than the breakthrough pressure, the liquid droplet will penetrate through the interstices and flood the surface. In order to design pillars that prevent surface flooding, the breakthrough pressure should be pre-determined to be higher than the total impacting pressure. Simplifying the problem for a liquid column under capillary action, the critical breakthrough pressure (*P_c_*) is defined as:

$$\begin{aligned} P_{c}=\frac{F_{c}}{A_{c}} \#\left( S SEQ ( \backslash* ARABIC 12 \right) \end{aligned}$$

*F_c_* is the capillary force (N), and $A_{c}$ is the capillary area (m). Assuming the capillary is balanced under the weight of the liquid and taking into account of the pillars’ overhang angle (relative to the interface), the capillary force is written as $F_{c}=\gamma_{lg}L_{c}\cos{(\theta_{e}-(\theta}_{Y}-90^{\circ}))$ where *L_c_* is the capillary perimeter (m), $\theta_{e}$ is the overhang angle (°) and $\gamma_{lg}$ is the liquid-gas interfacial surface tension (N/m). Inserting the capillary force into the breakthrough pressure equation above gives [7,8]:

$$\begin{aligned} P_{c}=\frac{\gamma_{lg}L_{c}\cos{(\theta_{e}-(\theta}_{Y}-90^{\circ}))}{A_{c}} \#\left( S SEQ ( \backslash* ARABIC 13 \right) \end{aligned}$$

By simplifying the problem for a unit pillar cell configuration (4 × 4), where the liquid forms a meniscus at the centre of impact, the dashed area as highlighted in **Figure S3** is described as the capillary area. Furthermore, the perimeter of the capillary can be determined from taking the total length of the meniscus along the contact lines of the solid-liquid and liquid-gas interfaces [7].

*
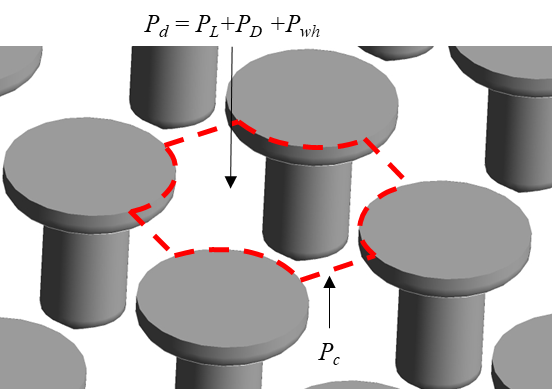
*

Figure S3. Doubly re-entrant unit cell array highlighting the liquid-solid interface of the capillary area during droplet impaction. The working pressures involve the Laplace pressure (*P_L_*), dynamic pressure (*P_D_*), water hammer pressure (*P_wh_*) and the breakthrough pressure (*P_c_*) [6,9].

The pressures as aforementioned in the diagram will be explained within the chapter. Notably, the capillary perimeter and area are distinctive to the geometry of the pillars. Firstly, for circular pillars in a rectangular pattern as a unit cell, the capillary perimeter is given as $L_{c}=\pi D$ with the capillary area being equal to $A_{c}=S^{2}-\frac{\pi D^{2}}{4}$ and the pillars’ overhang angle equal to 90°. Substituting into the breakthrough pressure equation gives [7,10]:

$$\begin{aligned} P_{c,p}=\frac{{4\pi D\gamma}_{lg}\cos\theta_{Y}}{4S^{2}-\pi D^{2}} \#\left( S SEQ ( \backslash* ARABIC 14 \right) \end{aligned}$$

where $P_{c,p}$ is the breakthrough pressure for circular straight pillars (Pa). For re-entrant pillars, the capillary perimeter and area are given to be the same as circular pillars, except that the overhang angle is equal to 0°. Therefore, the breakthrough pressure for re-entrant pillars, $P_{c,re}$, is given as:

$$\begin{aligned} P_{c,re}=\frac{{4\pi D\gamma}_{lg}\sin\theta_{Y}}{4S^{2}-\pi D^{2}} \#\left( S SEQ ( \backslash* ARABIC 15 \right) \end{aligned}$$

Importantly, however, this equation is only valid if *θ_Y_* ≤ 90°. To explain why, there are two main wetting phenomena that occur when pillars are under capillary action. These are known as the Canthotaxis and Laplace breakthrough effect. The Canthotaxis effect states that as the hydrostatic pressure of the fluid increases through the capillaries, the apparent contact angle of the meniscus begins to increase up to a critical value where the three-phase contact line (initially pinned at the pillars’ edge) begins to slide downwards along the sidewalls. This critical apparent contact angle is given as [11]:

$$\begin{aligned} \theta_{max}= \theta_{Y}+(180^{\circ}-\theta_{e})\#\left( S SEQ ( \backslash* ARABIC 16 \right) \end{aligned}$$

As a result, partial wetting (where the liquid wicks the cavities but does not wet the projected area) or homogenous wetting may occur from this effect [12]. On the other hand, the Laplace breakthrough effect is given when the pressure difference between the droplet and the capillary is greater than the breakthrough pressure before reaching the maximum apparent contact angle. This causes the meniscus to bulge further into the cavity whilst the three-phase contact line remains to be pinned. As a consequence, the penetration depth of the liquid-gas interface is now dependent on the surface tension force and the pitch of the interstice. To summarise, two boundary conditions can be derived to determine the stability of the wetting state: if $\theta_{Y}\leq\theta_{e}+90^{\circ}$, the Canthotaxis effect will take place; but if $\theta_{Y}>\theta_{e}+90^{\circ}$, the Laplace breakthrough effect will occur [8]. Regarding the breakthrough pressure equation for re-entrant pillars, if the *θ_Y_* > 90°, the Laplace breakthrough effect will take place therefore reducing the equation to:

$$\begin{aligned} P_{c,re}=\frac{{4\pi D\gamma}_{lg}}{4S^{2}-\pi D^{2}} \#\left( S SEQ ( \backslash* ARABIC 17 \right) \end{aligned}$$

Furthermore, for doubly re-entrant pillars, the capillary perimeter and area are slightly altered from circular pillars giving $L_{c}=\pi D^{'}$ and $A_{c}=S^{2}-\frac{\pi D^{'2}}{4}$. Here, $D^{'}$ is given as $D^{'}=D-2t_{o}$. As the overhang angle is determined to be -90°, the Laplace breakthrough effect will always take place for any intrinsic contact angle (as $\theta_{Y}>0^{\circ}$) from the Laplace breakthrough condition. Therefore, the breakthrough pressure for doubly re-entrant pillars, $P_{c,dre}$, is given as:

$$\begin{aligned} P_{c,dre}=\frac{{4\pi D^{'}\gamma}_{lg}}{{4S}^{2}-\pi D^{'2}} \#\left( S SEQ ( \backslash* ARABIC 18 \right) \end{aligned}$$

For the case of a sessile droplet placed on a composite interface, the breakthrough pressure only needs to, ideally, overcome the Laplace pressure which is generally stated as:

$$\begin{aligned} P_{L}=\frac{2\gamma_{lg}}{R} \#\left( S SEQ ( \backslash* ARABIC 19 \right) \end{aligned}$$

For millimetric sized droplets, the effect of the Laplace pressure can be considered to be negligible. Nonetheless, for impacting droplets, additional external pressures are involved, namely, the dynamic pressure derived from Bernoulli’s principle $(P_{D}=\frac{1}{2}\rho_{l}V^{2})$ and the water hammer (or shock) pressure. The water hammer pressure is essentially the pressure due to the rapid deacceleration of a liquid which is given as [13]:

$$\begin{aligned} P_{wh}=k\rho_{l}cV \#\left( S SEQ ( \backslash* ARABIC 20 \right) \end{aligned}$$

where *k* is the water hammer pressure coefficient and *c* is the speed of sound within the liquid (m/s) [9,14]. Notably, the water hammer pressure coefficient is mainly determined experimentally which is based on the critical impact velocity that allows the droplet to transition from a non-wetting to a wetting state [9]. However, the coefficient can be estimated based on the pressure balance equation between the breakthrough pressure and the dynamic pressures involved with $P_{c}=P_{D}+P_{wh}$. Re-arranging the equation for the shock pressure coefficient gives [9]:

$$\begin{aligned} k=\frac{P_{c}-P_{d}}{\rho_{l}cV} \#\left( S SEQ ( \backslash* ARABIC 21 \right) \end{aligned}$$

In summary, to prevent the impacting droplet from either partially or fully wetting the composite interface, the net pressure, that is, the total impacting pressure proportional to the droplets’ size and velocity must be less than the breakthrough pressure generated from the pillars’ dimensions and design ($P_{c}>P_{D}+P_{wh}+P_{L}$).

**6. Droplet impact videos**

The droplet impact process was experimentally imaged using a high-speed camera (Photron, FASTCAM Mini UX100; image analysis software PFV4). The frame rate of the high-speed camera was 2000 fps with a resolution of up to 1280 x 1024. The volume of the droplet was matched to the value used for the simulations. The height between the syringe that delivered the droplet and the top of the sample surface was adjusted to create a free fall velocity of 0.3-0.34 m/s. All videos shown are rendered at 15 fps for ease of viewing.

**Video S1.** Simulated droplet impact on doubly re-entrant pillars having a pitch size (S) of 150 µm with an intrinsic contact angle (*θ_Y_*) of 70°.

**Video S2.** Experimental droplet impact on untreated doubly re-entrant pillars having a pitch size (S) of 150 µm.

**Video S3.** Simulated droplet impact on doubly re-entrant pillars having a pitch size (S) of 150 µm with an intrinsic contact angle (*θ_Y_*) of 120°.

**Video S4.** Experimental droplet impact on hydrophobized doubly re-entrant pillars having a pitch size (S) of 150 µm.

**Video S5.** Simulated droplet impact on doubly re-entrant pillars having a pitch size (S) of 200 µm with an intrinsic contact angle (*θ_Y_*) of 70°.

**Video S6.** Experimental droplet impact on untreated doubly re-entrant pillars having a pitch size (S) of 200 µm.

**Video S7.** Simulated droplet impact on doubly re-entrant pillars having a pitch size (S) of 200 µm with an intrinsic contact angle (*θ_Y_*) of 120°.

**Video S8.** Experimental droplet impact on hydrophobized doubly re-entrant pillars having a pitch size (S) of 200 µm.

**Video S9.** Simulated droplet impact on doubly re-entrant pillars having a pitch size (S) of 250 µm with an intrinsic contact angle (*θ_Y_*) of 70°.

**Video S10.** Experimental droplet impact on untreated doubly re-entrant pillars having a pitch size (S) of 250 µm.

**Video S11.** Simulated droplet impact on doubly re-entrant pillars having a pitch size (S) of 250 µm with an intrinsic contact angle (*θ_Y_*) of 120°.

**Video S12.** Experimental droplet impact on hydrophobized doubly re-entrant pillars having a pitch size (S) of 250 µm.

**Video S13.** Simulated droplet impact on re-entrant pillars with an intrinsic contact angle (*θ_Y_*) of 70°

**Video S14.** Simulated droplet impact on circular straight pillars with an intrinsic contact angle (*θ_Y_*) of 70°

**Video S15.** Simulated droplet impact on doubly re-entrant pillars with D=100 µm and *θ_Y_* = 105°.

**Video S16.** Simulated droplet impact on doubly re-entrant pillars with D = 150 µm and *θ_Y_* = 105°.

**Video S17.** Simulated droplet impact on doubly re-entrant pillars with D = 200 µm and *θ_Y_* = 105°.

**Video S18.** Simulated droplet impact on doubly re-entrant pillars with H = 50 µm and *θ_Y_* = 105°.

**Video S19.** Simulated droplet impact on doubly re-entrant pillars with H = 30 µm and *θ_Y_* = 105°.

**Video S20.** Simulated droplet impact on doubly re-entrant pillars with δ = 15 µm and *θ_Y_* = 105°.

**Video S21.** Simulated droplet impact on doubly re-entrant pillars with δ = 5 µm and *θ_Y_* = 105°.

**Video S22.** Simulated droplet impact on doubly re-entrant pillars with t_o_ = 15 µm and *θ_Y_* = 105°.

**Video S23.** Simulated droplet impact on doubly re-entrant pillars with t_o_ = 5 µm and *θ_Y_* = 105°.

**Video S24.** Simulated droplet impact on doubly re-entrant pillars with S = 200 µm and *θ_Y_* = 105°.

**Video S25.** Simulated droplet impact on doubly re-entrant pillars with S = 250 µm and *θ_Y_* = 105°.

**7. Solid fraction of doubly re-entrant pillars**

From **Figure S3**, the interfacial area is the sum of the planar cross-sectional area and the surface area of the vertical overhang $\pi D\delta$/4 (neglecting t_0_) [7]. The calculation of the solid fraction is given below:

$$\begin{aligned} \varphi_{s}=\frac{\left( \frac{\pi D^{2}}{16}+\frac{\pi D\delta}{4} \right)\times4}{S^{2}} \\ \varphi_{s}=\frac{\pi D^{2}+4\pi D\delta}{4S^{2}}\#\left( S SEQ ( \backslash* ARABIC 22 \right) \end{aligned}$$

**8. Interfacial pressure, penetration depth and spreading diameter**

**Figure S4** displays the wetting area, interfacial pressure and penetration height profiles of the droplet impacting the configurated pillars by varying the width between 100 to 200 µm. As expected, increasing the width causes the maximum and minimum wetting diameter to increase as the droplet struggles to recede once it is fully spread. **Figure S5** shows the spreading diameter, interfacial pressure, and penetration depth profiles for each of the pitch variations from 150 to 250 µm. The effects of the pillar height against wettability are displayed in **Figure S6.** To explore the effects of the length of the overhang on droplet repellency of the surface, **Figure S7** demonstrates the wetting diameter, interfacial pressure, and droplet penetration profiles against various overhang lengths. **Figure S8** depicts the contact area, interfacial pressure and penetration depth of the droplet for each of the overhang thickness values. **Figure S9** displays the spreading factor results against contact time for various pitch, height and diameter sizes with an intrinsic contact angle of 105°. The spreading factor (*β*) is defined as the ratio of the maximum spreading diameter to the diameter of the droplet ($\beta=\frac{\lambda_{max}}{\lambda_{c}})$.

**
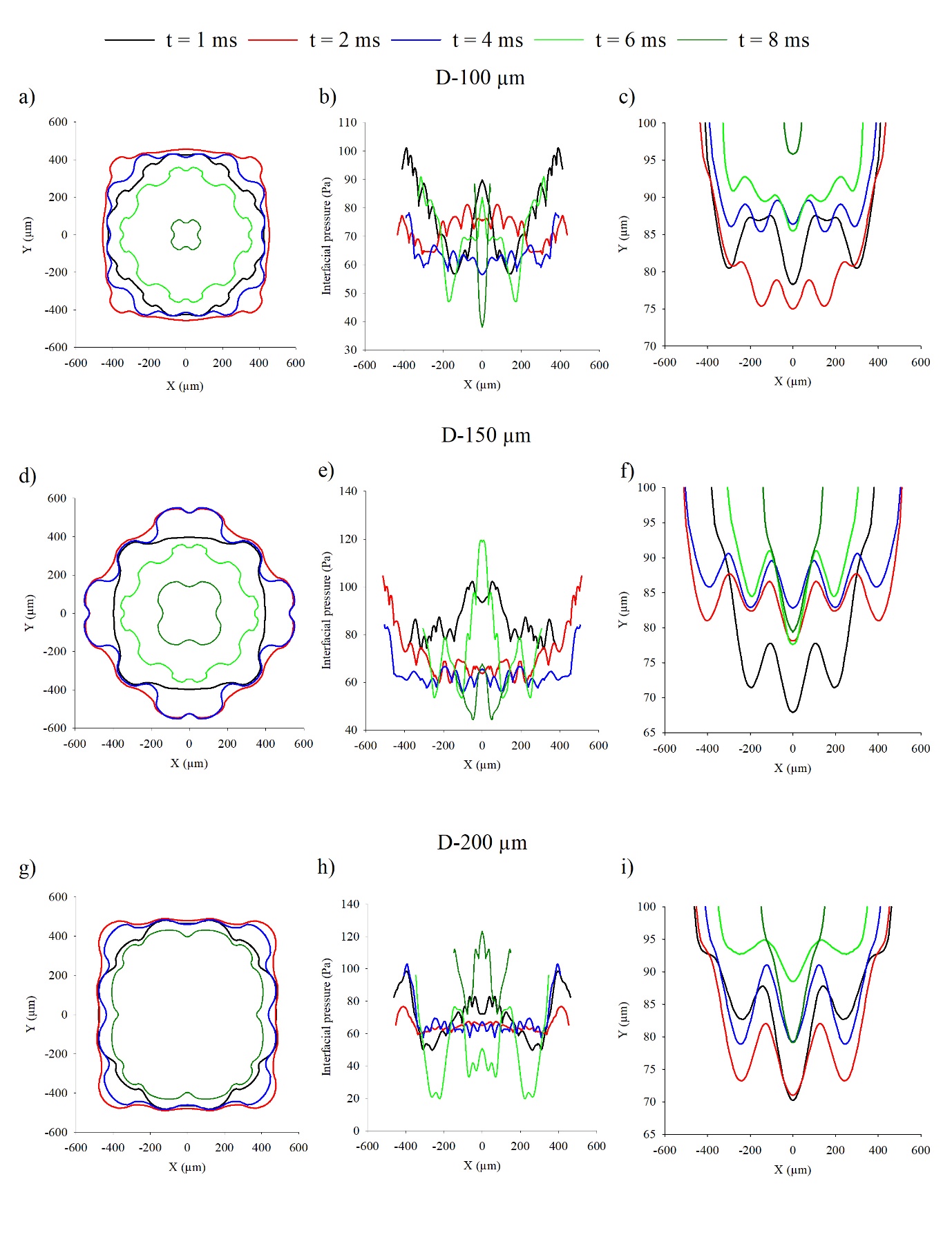
**

Figure S4. Plots of contact area (a, d and g), interfacial pressure (b, e and h) and penetration depth (c, f and i) of droplet impacting configurated doubly re-entrant pillars at different timescales for an *θ_Y_* = 105°. (a-c): D=100 µm. (d-f): D=150 µm. (g-i): D=200 µm.


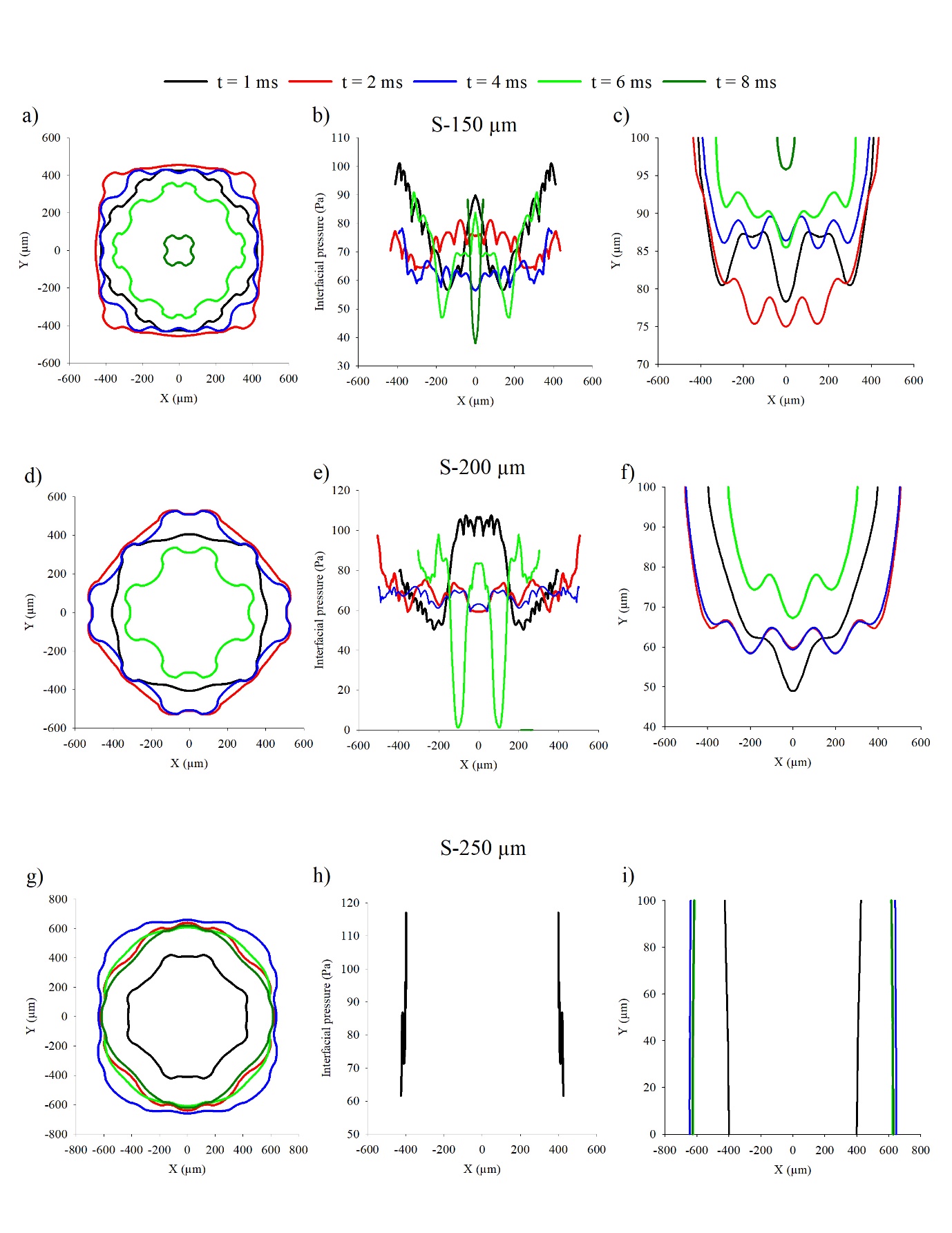


Figure S5. Plots of contact area (a, d and g), interfacial pressure (b, e and h) and penetration depth (c, f and i) of droplet impacting configurated doubly re-entrant pillars at different timescales for *θ_Y_* = 105°. (a-c): S=150 µm. (d-f): S=200 µm. (g-i): S=250 µm.


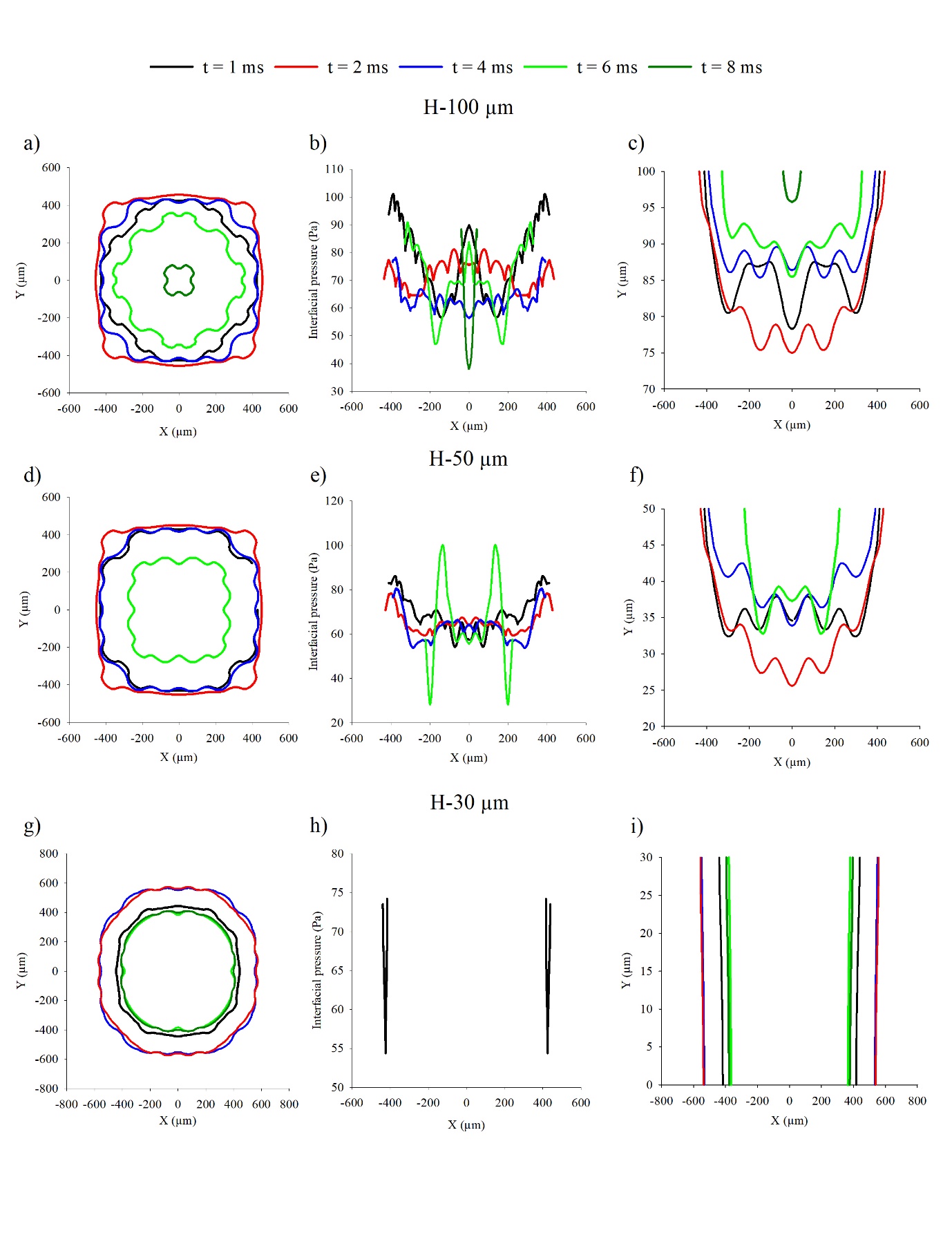


Figure S6. Plots of contact area (a, d and g), interfacial pressure (b, e, and h) and penetration depth (c, f and i) of droplet impacting configurated doubly re-entrant pillars at different timescales for *θ_Y_* = 105°. (a-c): H=100 µm. (d-f): H=50 µm. (g-i): H=30 µm.


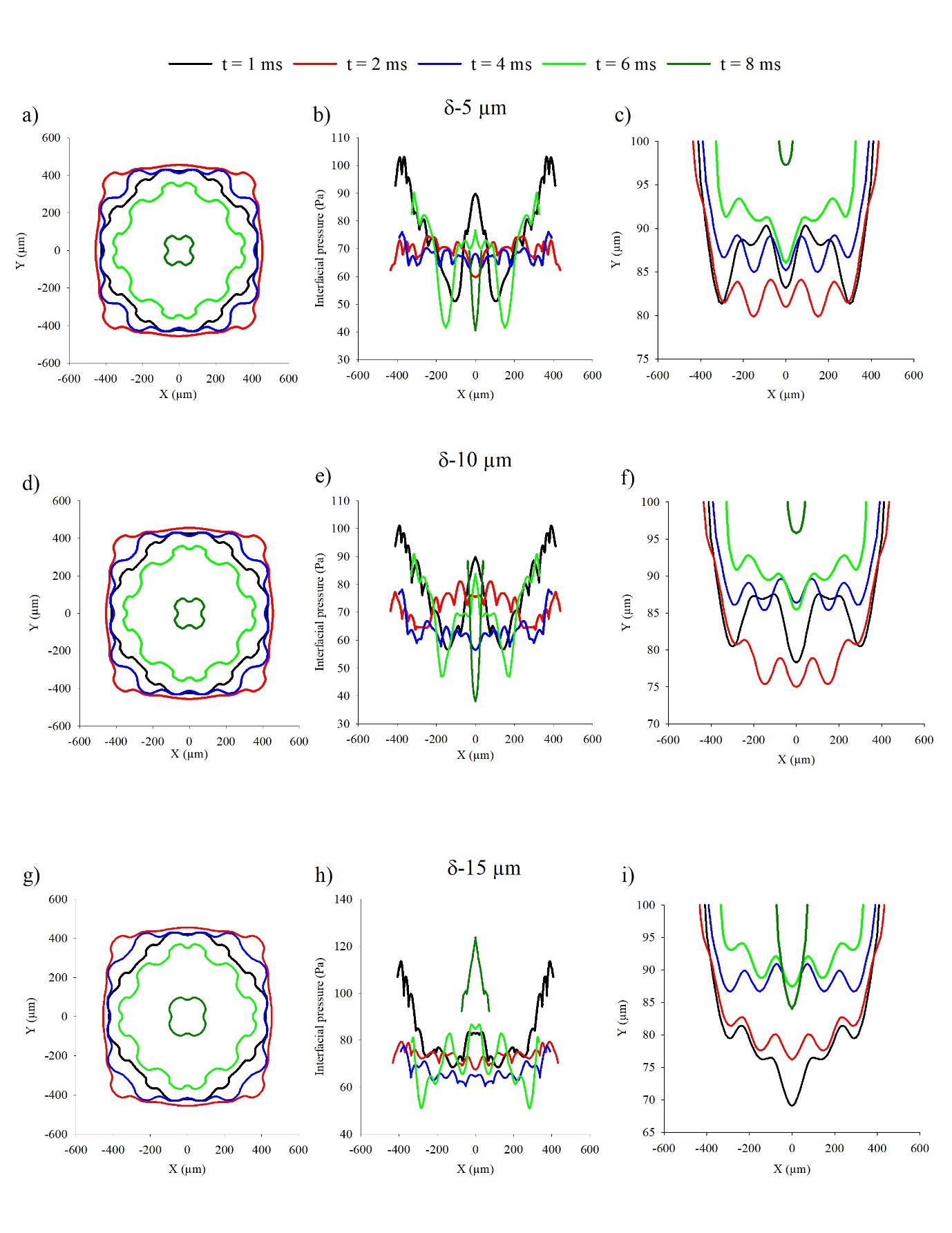


Figure S7. Plots of contact area (a, d and g), interfacial pressure (b, e and h) and penetration depth (c, f and i) of droplet impacting configurated doubly re-entrant pillars at different timescales for *θ_Y_* = 105°. (a-c): δ=5 µm. (d-f): δ=10 µm. (g-i): δ=15 µm.


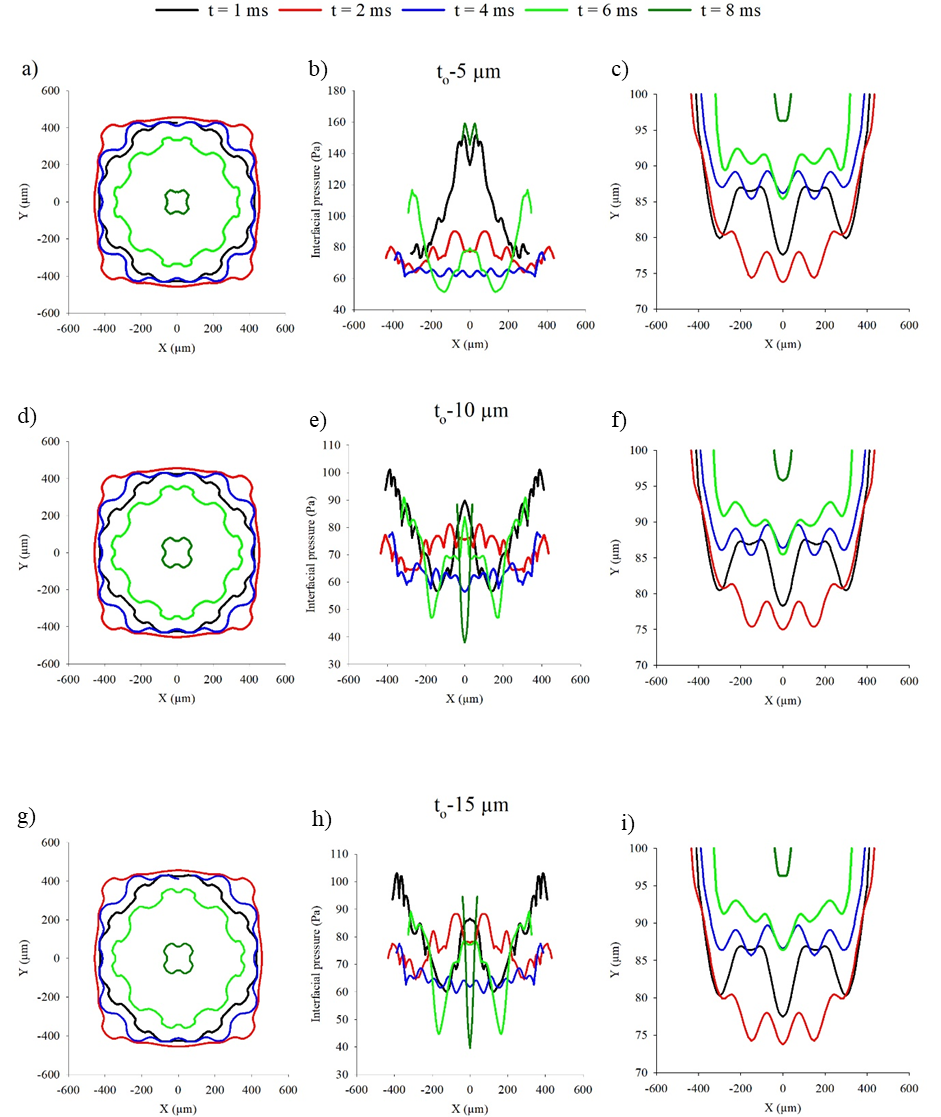


Figure S8. Plots of contact area (a, d and g), interfacial pressure (b, e and h) and penetration depth (c, f and i) of droplet impacting configurated doubly re-entrant pillars at different timescales for *θ_Y_* = 105°. (a-c): t_o_=5 µm. (d-f): t_o_=10 µm. (g-i): t_o_=15 µm.


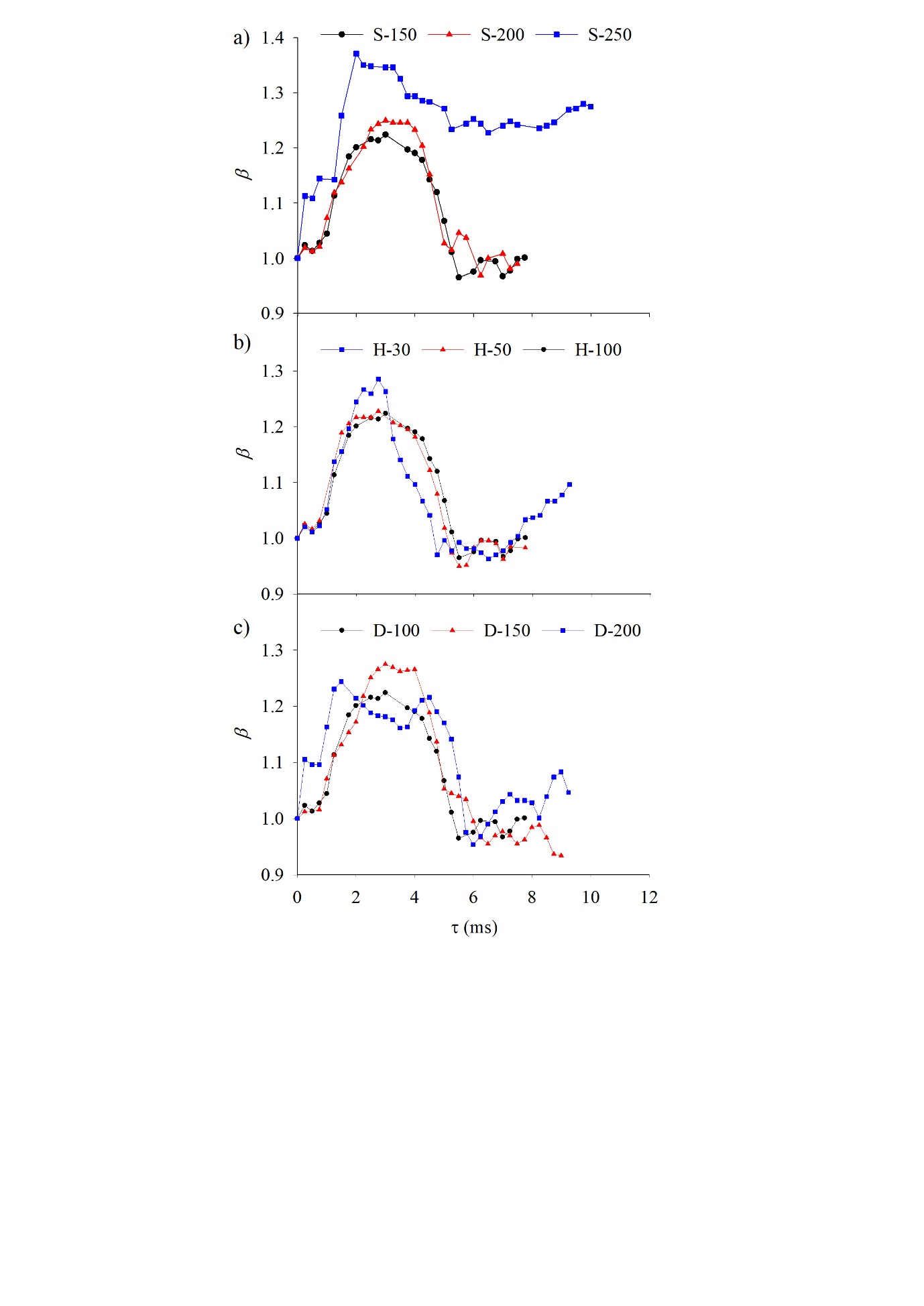


Figure S9. Spreading factor against contact time for various dimensional sizes of the doubly re-entrant pillars with an intrinsic contact angle of *θ_Y_* = 105°. (a): pitch spacing. (b): height. (c): diameter.

**References**

1 Brackbill, J. U., Kothe, D. B. & Zemach, C. A continuum method for modeling surface tension. *J. Comput. Phys.* **100**, 335-354 (1992).

2 Ubbink, O. Numerical prediction of two fluid systems with sharp interfaces. (1997).

3 Johnsen, E. Spurious oscillations and conservation errors in interface-capturing schemes. *CTR Ann. Res. Briefs*, 115-126 (2008).

4 Tatekura, Y., Watanabe, M., Kobayashi, K. & Sanada, T. Pressure generated at the instant of impact between a liquid droplet and solid surface. *R. Soc. Open Sci.* **5**, 181101 (2018).

5 Courant, R., Friedrichs, K. & Lewy, H. On the partial difference equations of mathematical physics. *IBM J. Res. Dev.* **11**, 215-234 (1967).

6 Hu, A. & Liu, D. 3D simulation of micro droplet impact on the structured superhydrophobic surface. *Int. J. Multiphase Flow* **147**, 103887 (2022).

7 Torkkeli, A. *Droplet microfluidics on a planar surface*. (VTT Technical Research Centre of Finland, 2003).

8 Berthier, J. *et al.* On the pinning of interfaces on micropillar edges. *J. Colloid Interface Sci.* **338**, 296-303 (2009).

9 Dash, S., Alt, M. T. & Garimella, S. V. Hybrid surface design for robust superhydrophobicity. *Langmuir* **28**, 9606-9615 (2012).

10 Liao, D., He, M. & Qiu, H. High-performance icephobic droplet rebound surface with nanoscale doubly reentrant structure. *Int. J. Heat Mass Transfer* **133**, 341-351 (2019).

11 Oliver, J., Huh, C. & Mason, S. Resistance to spreading of liquids by sharp edges. *J. Colloid Interface Sci.* **59**, 568-581 (1977).

12 Hensel, R. *et al.* Wetting resistance at its topographical limit: the benefit of mushroom and serif T structures. *Langmuir* **29**, 1100-1112 (2013).

13 Maitra, T. *et al.* On the nanoengineering of superhydrophobic and impalement resistant surface textures below the freezing temperature. *Nano Lett.* **14**, 172-182 (2014).

14 Hee Kwon, D. & Joon Lee, S. Impact and wetting behaviors of impinging microdroplets on superhydrophobic textured surfaces. *Appl. Phys. Lett.* **100**, 171601 (2012).
